# Mocetinostat activates Krüppel-like factor 4 and protects against tissue destruction and inflammation in osteoarthritis

**DOI:** 10.1172/jci.insight.170513

**Published:** 2023-09-08

**Authors:** Manabu Kawata, Daniel B. McClatchy, Jolene K. Diedrich, Merissa Olmer, Kristen A. Johnson, John R. Yates, Martin K. Lotz

**Affiliations:** 1 Department of Molecular Medicine, Scripps Research, La Jolla, California, USA.; 2 Calibr, a Division of Scripps Research, La Jolla, California, USA.

**Keywords:** Bone Biology, Osteoarthritis

## Abstract

Osteoarthritis (OA) is the most common joint disorder, and disease-modifying OA drugs (DMOADs) represent a major need in OA management. Krüppel-like factor 4 (KLF4) is a central transcription factor upregulating regenerative and protective functions in joint tissues. This study was aimed to identify small molecules activating KLF4 expression and to determine functions and mechanisms of the hit compounds. High-throughput screening (HTS) with 11,948 clinical-stage compounds was performed using a reporter cell line detecting endogenous KLF4 activation. Eighteen compounds were identified through the HTS and confirmed in a secondary screen. After testing in SW1353 chondrosarcoma cells and human chondrocytes, mocetinostat — a class I selective histone deacetylase (HDAC) inhibitor — had the best profile of biological activities. Mocetinostat upregulated cartilage signature genes in human chondrocytes, meniscal cells, and BM-derived mesenchymal stem cells, and it downregulated hypertrophic, inflammatory, and catabolic genes in those cells and synoviocytes. I.p. administration of mocetinostat into mice reduced severity of OA-associated changes and improved pain behaviors. Global gene expression and proteomics analyses revealed that regenerative and protective effects of mocetinostat were dependent on peroxisome proliferator-activated receptor γ coactivator 1-α. These findings show therapeutic and protective activities of mocetinostat against OA, qualifying it as a candidate to be used as a DMOAD.

## Graphical abstract

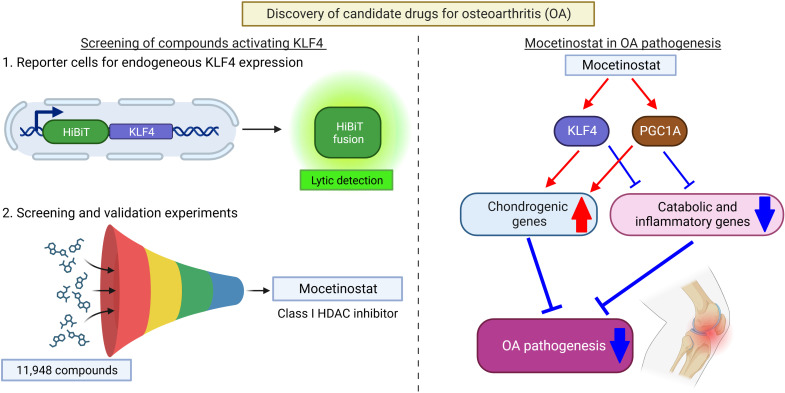

## Introduction

Osteoarthritis (OA) is the most prevalent joint disease, and the burden of OA is becoming a major global socioeconomic problem due to population aging (1). Despite substantial efforts, there have been no approved disease-modifying OA drugs (DMOADs) (2).

The Krüppel-like factor (KLF) family of transcription factors is composed of 17 members in mammals and is involved in various biological and pathological mechanisms (3). We previously found that expression of KLF4 is suppressed with OA progression and aging in human and mouse knee cartilage and that it has a spectrum of biological activities as a promising therapeutic target of OA (4, 5). KLF4 enhances expression of SRY-box transcription factor-9 (SOX9) and major cartilage extracellular matrix (ECM) genes including type 2 and type 11 collagen (COL2A1 and COL11A2, respectively), aggrecan (ACAN), cartilage oligomeric matrix protein (COMP), and proteoglycan-4 (PRG4), in human chondrocytes, meniscal cells, and BM-derived mesenchymal stem cells (BMSCs) (5). Meanwhile, KLF4 suppresses mediators of inflammation and ECM-degrading enzymes, such as a disintegrin and metalloproteinase with thrombospondin motifs-5 (ADAMTS5), matrix metalloproteinase-3 (MMP3), MMP13, IL-6, and prostaglandin-endoperoxide synthase-2 (PTGS2), in human chondrocytes, meniscal cells, and synoviocytes (5). Furthermore, viral KLF4 delivery into mouse knees has therapeutic and protective effects against OA-associated tissue damage and pain (5). Considering all the features of KLF4, we hypothesized that molecules increasing KLF4 expression would be novel therapeutic candidates for OA, consistent with the general concept of therapeutic targeting transcription factors in OA (6).

HiBiT is a 1.3 kDa peptide (11 amino acids) capable of producing bright and quantitative luminescence, and when integrated into the genome, it serves as an efficient reporter tag for endogenous proteins in high-throughput screening (HTS) of drugs (7).

Here, we applied HiBiT to HTS of small molecules activating endogenous KLF4 expression to discover potentially novel candidates of DMOADs, and we identified mocetinostat, a class I selective histone deacetylase (HDAC) inhibitor. Furthermore, we analyzed functions of mocetinostat in human joint tissue cells and in a mouse OA model, and we determined mechanisms by which mocetinostat protects against OA-associated damage.

## Results

### HTS of small molecules activating endogenous KLF4 expression.

**Figure 1:**
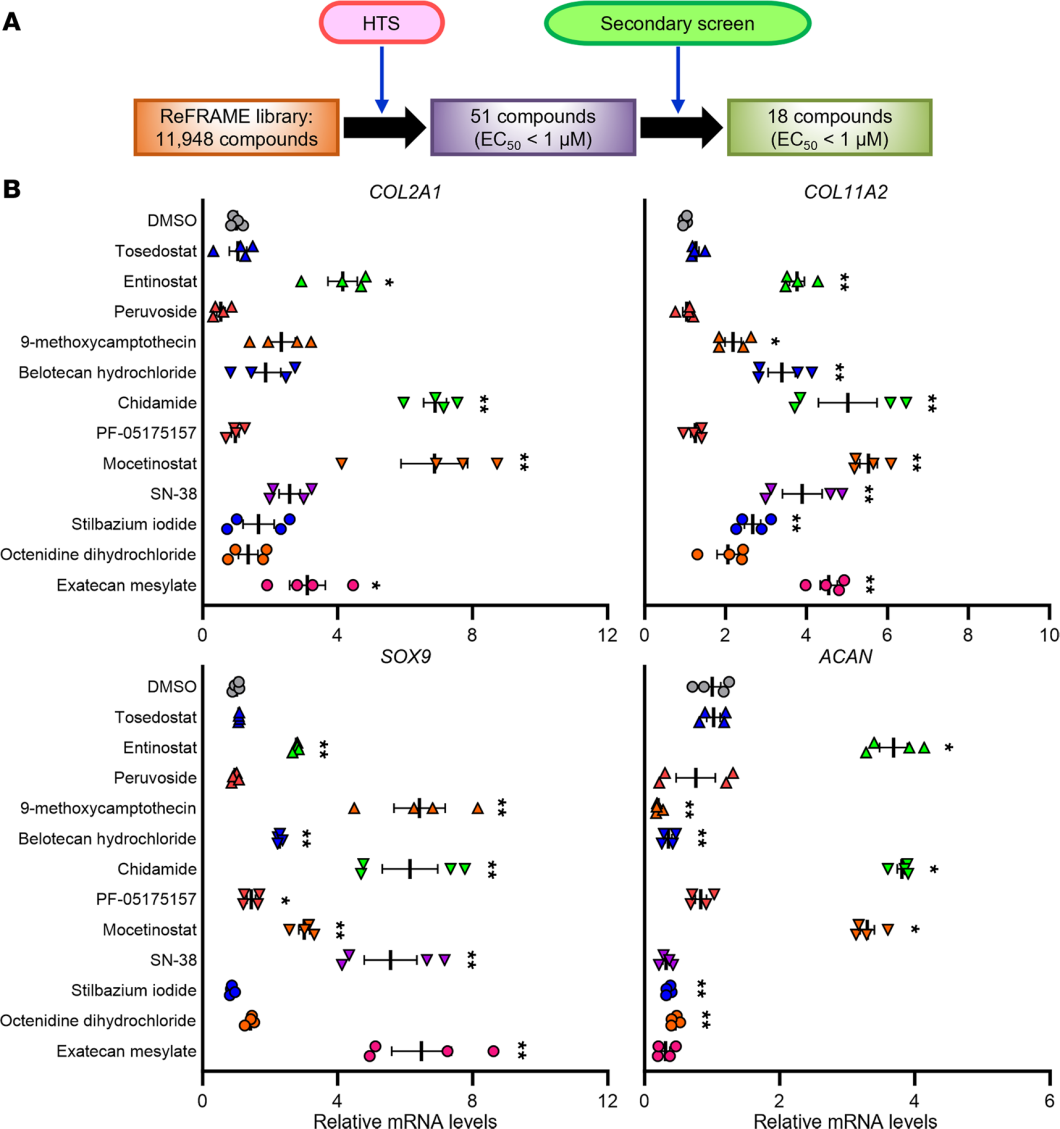
Screens of compounds activating *KLF4* expression and validation experiments with SW1353 cells. (**A**) A summary for the high-throughput screening (HTS) and the secondary screen. (**B**) Treatment of SW1353 cells with the compounds upregulating *KLF4*. Cells were treated with either of the compounds upregulating *KLF4* at the tolerated doses or dimethyl sulfoxide (DMSO), and RNA was collected 24 hours after initiation of treatment. mRNA levels are expressed as mean ± SEM, relative to DMSO (*n* = 4 from 4 independent experiments). *P < 0.05, **P < 0.01, Dunnett’s test versus DMSO. Results of 1-way mixed-effects ANOVA test are shown in Supplemental Table 17.

We established a KLF4 reporter cell line from SW1353 human chondrosarcoma cells, which enabled detection of endogenous KLF4 activation by HiBiT luminescence (Supplemental Figure 1; supplemental material available online with this article; https://doi.org/10.1172/jci.insight.170513DS1; also see Methods for details). Then, we performed HTS using the Repurposing, Focused Rescue, and Accelerated Medchem (ReFRAME) library, which is composed of 11,948 small molecules that have reached clinical development or undergone significant preclinical profiling (8, 9). Fifty-one hit compounds with EC_50_ < 1 μM were identified in the HTS, and 18 compounds were confirmed in a secondary screen (Figure 1A and Supplemental Tables 1 and 2).

### Validation experiments for the identified compounds.

We treated SW1353 cells with different doses of the 18 confirmed hit compounds to determine tolerated doses, as measured by cell viability (Supplemental Table 3). Twelve compounds were confirmed to significantly upregulate *KLF4* expression with the tolerated doses, as compared with dimethyl sulfoxide (DMSO) treatment (Supplemental Figure 2). We further measured expression levels of representative chondrogenic genes in the cells treated with either of the 12 compounds upregulating *KLF4* at the tolerated doses and found that 3 class I HDAC inhibitors (entinostat, chidamide, and mocetinostat) significantly increased expression of all the cartilage signature genes, *COL2A1*, *COL11A2*, *SOX9*, and *ACAN* (Figure 1B and Supplemental Table 4).

**Figure 2:**
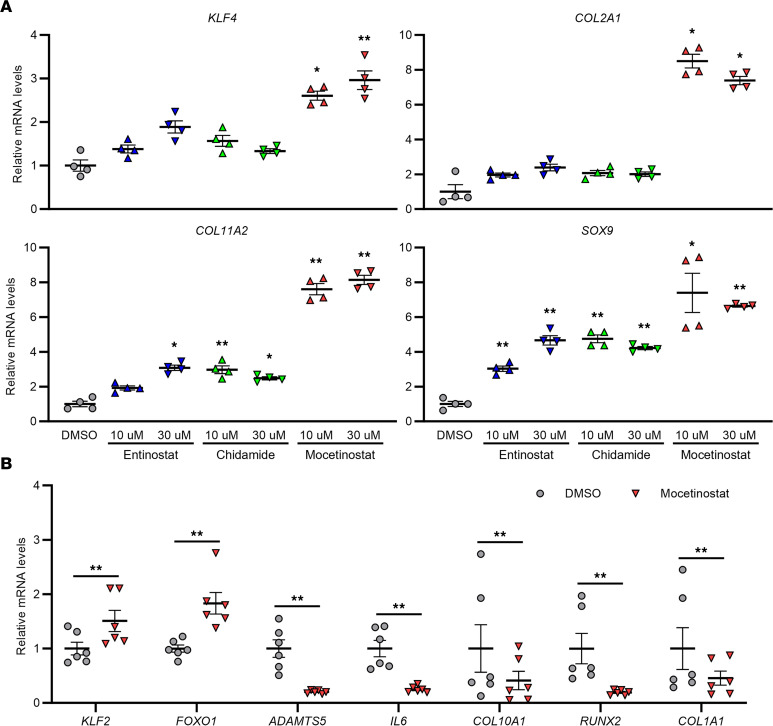
Treatment of human OA chondrocytes with the class I HDAC inhibitors. (**A**) Cells were treated with entinostat, chidamide, mocetinostat, or DMSO, and RNA was collected 24 hours after initiation of treatment. mRNA levels are expressed as mean ± SEM, relative to DMSO (*n* = 4 donors). *P < 0.05, **P < 0.01, Dunnett’s test versus DMSO. Results of 1-way mixed-effects ANOVA test are shown in Supplemental Table 17. (**B**) Cells were treated with 30 μM of mocetinostat or DMSO, and RNA was collected 24 hours after initiation of treatment. mRNA levels are expressed as mean ± SEM, relative to DMSO (*n* = 6 donors). **P < 0.01, paired *t* test. HDAC, histone deacetylase.

When human OA chondrocytes were treated with the 3 class I HDAC inhibitors, only mocetinostat significantly upregulated *KLF4*, and it also increased expression levels of *COL2A1*, *COL11A2*, and *SOX9* (Figure 2A). While mocetinostat upregulated *KLF2*, another KLF family member with functions similar to KLF4 (5), it also increased expression of forkhead box O1 (*FOXO1*) and downregulated *ADAMTS5* and *IL6* (Figure 2B); these effects are similar to those of KLF4 (5). Furthermore, mocetinostat suppressed mRNA levels of the chondrocyte hypertrophy markers, *COL10A1* and runt-related transcription factor 2 (*RUNX2*), and the fibroblast marker *COL1A1* (Figure 2B). Based on these biological activities of mocetinostat, we focused subsequent studies on this compound.

### Functions of mocetinostat in human BMSCs and joint tissue cells.

**Figure 3:**
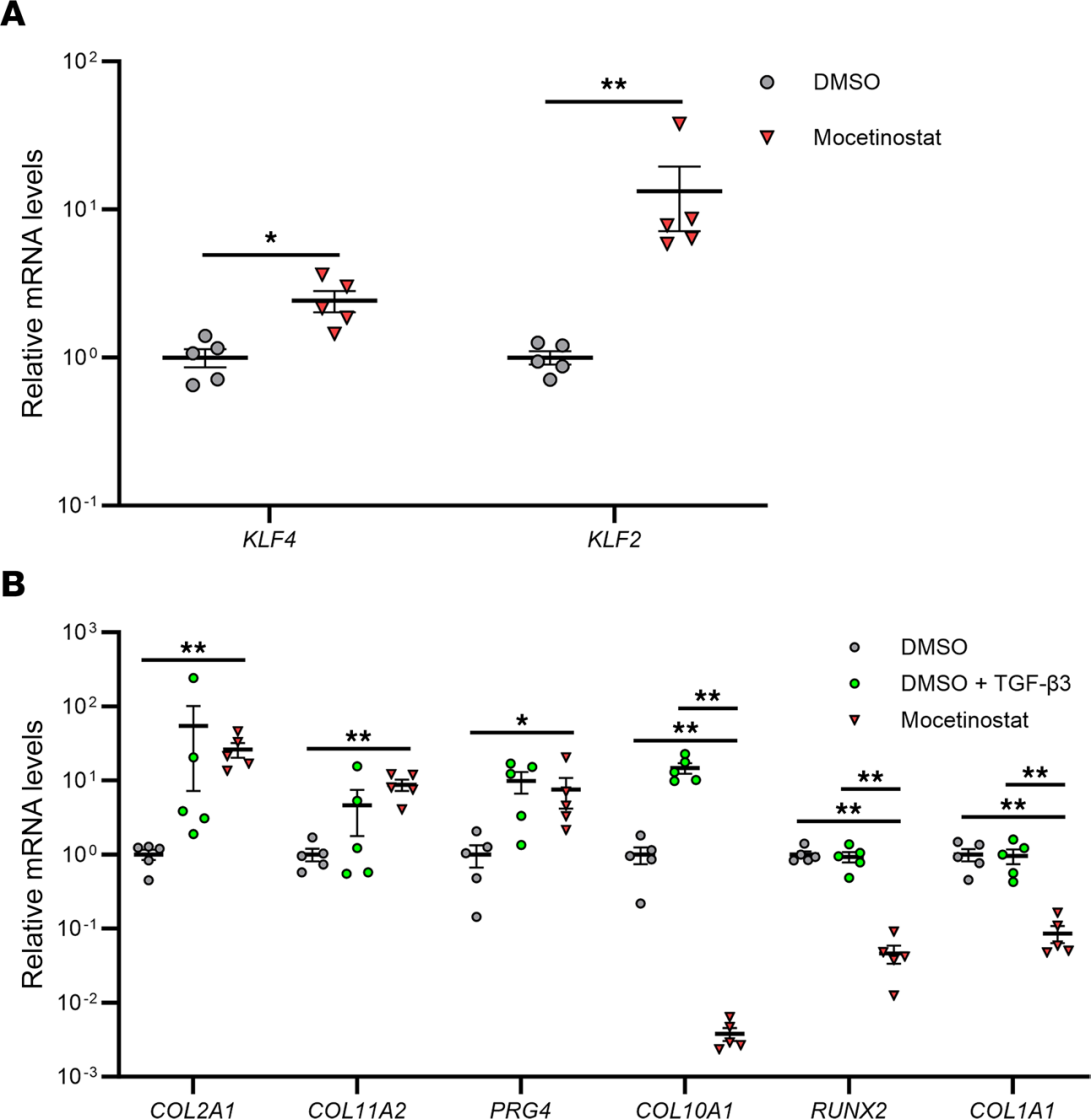
Regulation of chondrogenic and hypertrophic genes by mocetinostat in human BMSC pellets. (**A** and **B**) Human BM-derived mesenchymal stem cells (BMSCs) were cultured in pellets and were treated with 5 μM of mocetinostat or DMSO ± 20 ng/mL of TGF-β3. RNA was collected 1 week after pellet culture. mRNA levels are expressed as mean ± SEM, relative to DMSO (*n* = 5 donors). *P < 0.05, **P < 0.01; paired *t* test in **A**, and Dunnett’s test versus mocetinostat in **B**. Results of 1-way mixed-effects ANOVA test are shown in Supplemental Table 17.

To further investigate functions of mocetinostat in regulation of joint tissue homeostasis, we tested it in several different cell types. During monolayer culture of human BMSCs, mocetinostat increased *KLF4*, *KLF2*, *COL2A1*, *COL11A2*, *PRG4*, and *SOX9* (Supplemental Figure 3). In pellet-cultured BMSCs, mocetinostat enhanced expression levels of *KLF4* and *KLF2*, and upregulated *COL2A1*, *COL11A2*, and *PRG4* comparable with TGF-β3, a known inducer of chondrogenesis in BMSC (Figure 3). Notably, mocetinostat treatment downregulated *COL10A1*, *RUNX2*, and *COL1A1* (Figure 3B). Human meniscal cells treated with mocetinostat showed higher mRNA levels of *KLF4*, *KLF2*, *COL2A1*, *COL11A2*, and *SOX9* (Supplemental Figure 4). It also upregulated scleraxis (*SCX*) and tenascin-B (*TNXB*), which are reported to be highly expressed in meniscus and are upregulated by KLF4 (5, 10).

**Figure 4:**
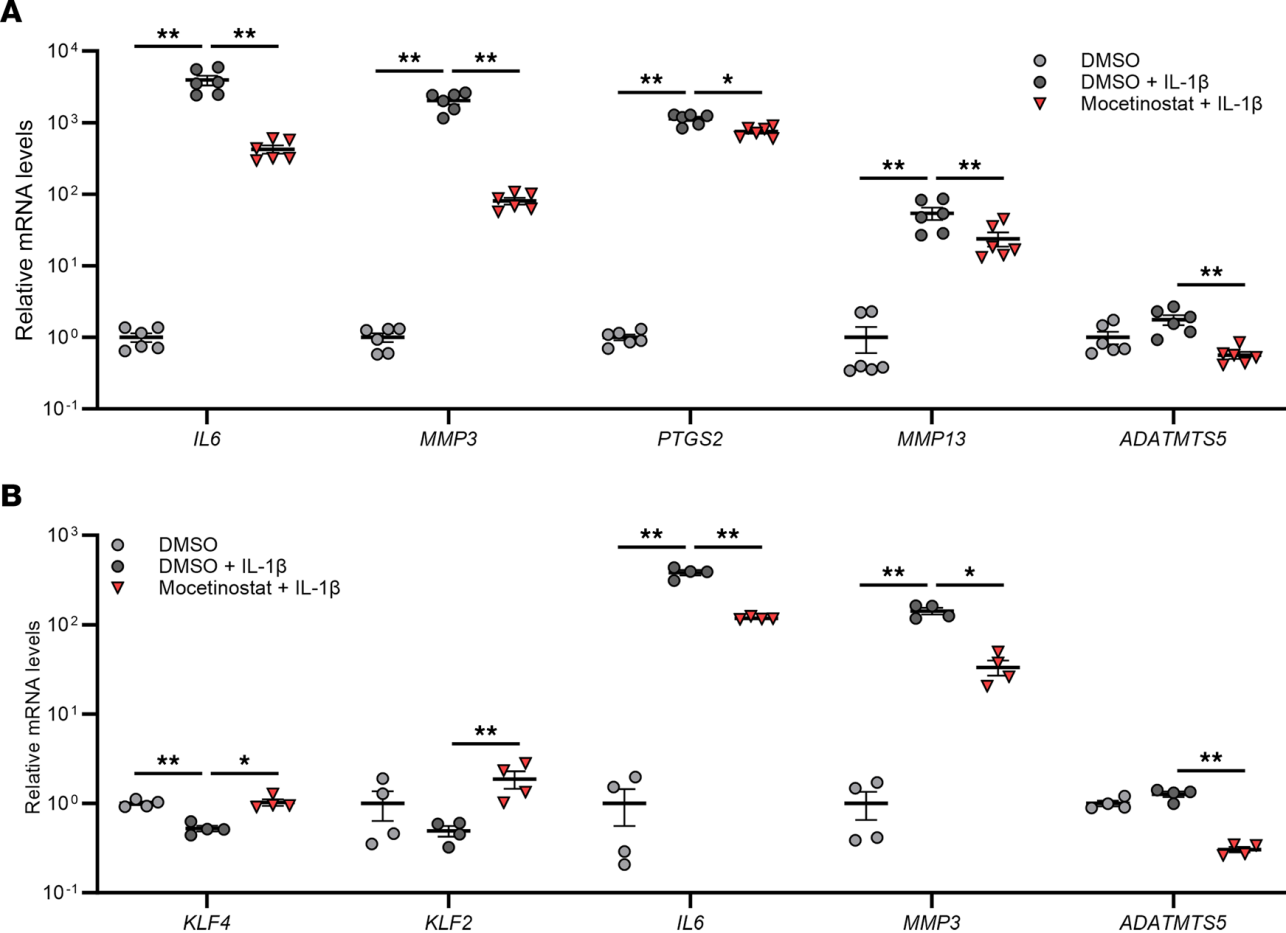
Regulation of inflammatory and catabolic genes by mocetinostat in human OA chondrocytes and synoviocytes on IL-1β stimulation. (**A**) Human OA chondrocytes were treated with 30 μM of mocetinostat or DMSO (*n* = 6 donors). (**B**) Human synoviocytes from healthy donors were treated with 10 μM of mocetinostat or DMSO (*n* = 4 donors). RNA was collected 24 hours after initiation of mocetinostat treatment and 6 hours after stimulation with 10 ng/mL of IL-1β. mRNA levels are expressed as mean ± SEM, relative to DMSO. *P < 0.05, **P < 0.01, Dunnett’s test versus DMSO + IL-1β. Results of 1-way mixed-effects ANOVA test are shown in Supplemental Table 17.

We examined regulation of genes related to inflammation and ECM degradation by mocetinostat. In human IL-1β–stimulated OA chondrocytes, mocetinostat significantly downregulated *IL6*, *MMP3*, *PTGS2*, *MMP13*, and *ADAMTS5* (Figure 4A). Similarly, mocetinostat suppressed *IL6*, *MMP3*, and *ADAMTS5* in human synoviocytes treated with IL-1β (Figure 4B). Collectively, mocetinostat enhanced chondrogenic and anabolic effects and also had antihypertrophic, antiinflammatory, and anticatabolic properties in joint tissue cells and BMSCs, suggesting its potential as a DMOAD.

### Therapeutic effects of mocetinostat in a mouse OA model.

**Figure 5:**
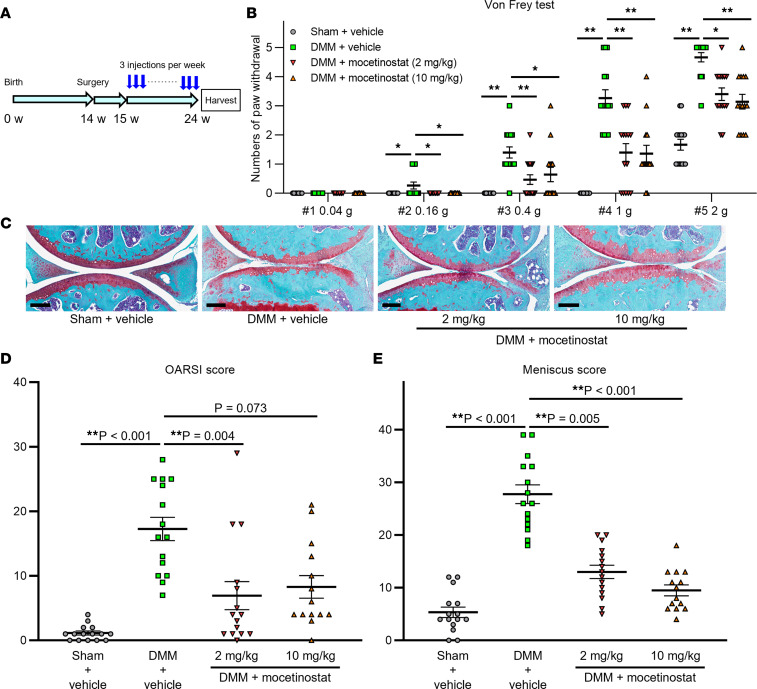
Therapeutic effects of mocetinostat in mouse OA model. (**A**) Fourteen-week-old mice underwent destabilization of the medial meniscus (DMM) or sham surgery, and mocetinostat or vehicle was injected i.p. 3 times a week starting 1 week after DMM surgery. Knees were harvested at 10 weeks postoperatively for histological analysis. (**B**) Results of von Frey test in mice at 10 weeks after surgery. Numbers of paw withdrawals from 5 stimulations per filament per mouse are shown. (**C**) Representative Safranin-O staining images for each group. Scale bars: 200 μm. (**D**) Summed Osteoarthritis Research Society International (OARSI) scores for the medial femoral condyle and the tibial plateau. (**E**) Meniscus histopathological scores. *n* = 14 for DMM + 10 mg/kg of mocetinostat, and *n* =15 for the other groups. For **B**, **D**, and **E**, *P < 0.05, **P < 0.01, Dunn’s test versus DMM + vehicle. All quantitative data are expressed as mean ± SEM, and results of Kruskal-Wallis test are shown in Supplemental Table 17.

To confirm therapeutic effects against OA in vivo, mice underwent OA induction by surgical destabilization of the medial meniscus (DMM) (11) and received i.p. injections of either 2 mg/kg or 10 mg/kg of mocetinostat 3 times per week starting 1 week after surgery, and their knee joints were harvested 10 weeks after surgery (Figure 5A). We performed von Frey test (12, 13) preoperatively and at 5 and 10 weeks after surgery to evaluate mechanical allodynia. Treatment with mocetinostat at both doses significantly decreased numbers of paw withdrawals at 5 and 10 weeks after DMM surgery, as compared with vehicle-injected mice (Figure 5B and Supplemental Figure 5). The severity of OA was significantly alleviated in the 2 mg/kg group and tended to be decreased in the 10 mg/kg group, as shown by the Osteoarthritis Research Society International (OARSI) scores (14) (Figure 5, C and D). Moreover, both 2 mg/kg and 10 mg/kg doses of mocetinostat significantly improved meniscus histopathological scores (15), synovitis scores (16), and bone scores (5, 17) (Figure 5E and Supplemental Figures 6 and 7). Bone scores (score, 0–8) were used to assess subchondral bone changes and ectopic ossification. There were no apparent systemic adverse reactions to mocetinostat injections, and no mice showed signs of distress requiring euthanasia. To determine whether these protective effects of mocetinostat were associated with increased KLF4 expression, we performed IHC for KLF4. Rates of KLF4^+^ cells were increased in cartilage from mice that received mocetinostat injections (Supplemental Figure 8). Meanwhile, expression of catabolic and inflammatory proteins, including ADAMTS5, IL-6, and MMP13, was significantly decreased in articular cartilage by mocetinostat treatment (Supplemental Figures 9–11). Furthermore, mocetinostat enhanced expression of FOXO1 (Supplemental Figure 12), which has protective functions in OA pathogenesis (17). These results demonstrated that mocetinostat increased KLF4 in vivo and reduced OA-associated joint damage and mechanical allodynia.

### Global analysis of genes regulated by mocetinostat.

**Figure 6:**
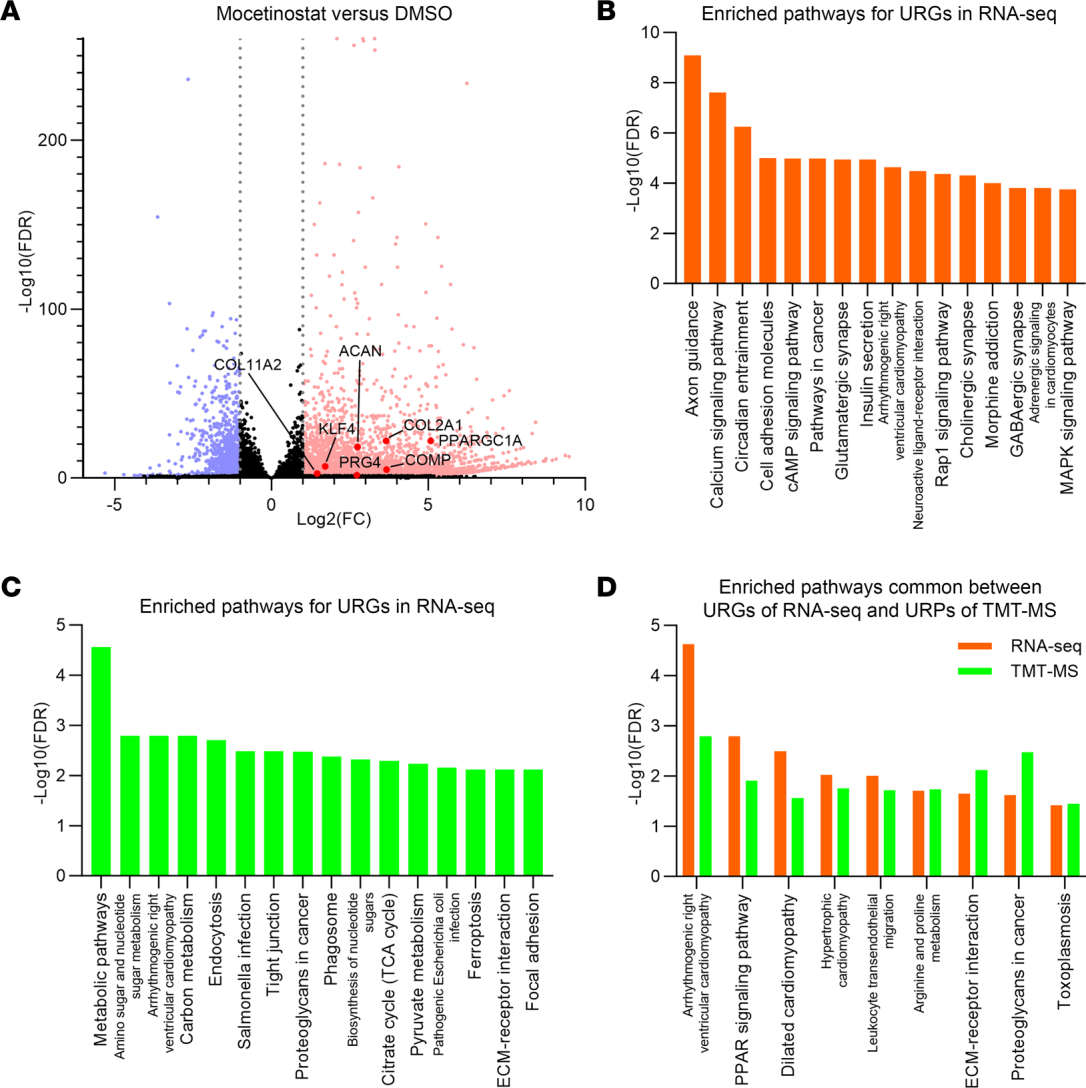
Global transcriptomics and proteomics analyses of mocetinostat- versus DMSO-treated TC28a2 cells. Cells were treated with 2 μM of mocetinostat or DMSO for 24 hours. *n* = 3 per condition from 3 independent experiments were analyzed by high-throughput RNA-Seq and tandem mass tag–mass spectrometry (TMT-MS). (**A**) Volcano plot of RNA-Seq data to identify differentially expressed genes (DEGs). Gray dotted lines indicate |log_2_(fold change [FC])| = 1. Significantly upregulated genes (URGs) are shown as red dots, while significantly downregulated genes (DRGs) are indicated as blue dots; black dots represent nonsignificant DEGs. (**B**) KEGG pathway analysis using URGs of RNA-Seq data. The top 16 enriched pathways are shown. (**C**) The top 16 enriched pathways in KEGG pathway analysis using significantly upregulated proteins (URPs) of TMT-MS data. (**D**) Enriched pathways common between URGs of RNA-Seq data and URPs of TMT-MS data.

To study genes regulated by mocetinostat comprehensively and to elucidate its regulatory mechanisms, we performed global expression profiling by high-throughput RNA-Seq analysis of mocetinostat- and DMSO-treated TC28a2 human chondrocyte cells. Lists of all significantly upregulated genes (URGs; 3,375 genes) and downregulated genes (DRGs; 1,171 genes) are shown in Supplemental Tables 5 and 6, where genes with a FDR < 0.05 and a |log_2_(fold change [FC])| > 1 were considered to be significantly differentially expressed. Mocetinostat significantly upregulated cartilage ECM genes such as *COL2A1*, *COL11A2*, *ACAN*, *COMP*, and *PRG4* (Figure 6A and Supplemental Table 5).

Kyoto Encyclopedia of Genes and Genomes (KEGG) pathway analyses (18) were performed using the URGs and DRGs. Significantly enriched pathways are shown in Figure 6B and Supplemental Tables 7 and 8. In the URGs, “ECM-receptor interaction,” where cartilage ECM genes such as *COL2A1* and *COMP* are annotated, was significantly enriched (Supplemental Table 7). While terms common with our previous data set using KLF4-overexpressing cells (5) — such as “Rap1 signaling pathway,” “Calcium signaling pathway,” “cAMP signaling pathway,” and “MAPK signaling pathway” — were also enriched (Figure 6B and Supplemental Table 7), enrichment of several unique pathways, including PPAR signaling pathway, was seen (Supplemental Table 7).

### Global proteomics analysis in mocetinostat-treated cells.

After confirming that mocetinostat increased KLF4 protein level (Supplemental Figure 13), we analyzed mocetinostat- and DMSO-treated TC28a2 cells using tandem mass tag–mass spectrometry (TMT-MS), and 5,036 proteins were quantified. Lists of all significantly upregulated proteins (URPs; 853 proteins) and downregulated proteins (DRPs; 1,035 proteins) are shown in Supplemental Tables 9 and 10, where proteins with an FDR < 0.05 were considered to be significantly differentially expressed. Significantly enriched pathways in KEGG pathway analysis using the URPs and DRPs are shown in Figure 6C and Supplemental Tables 11 and 12.

When we intersected significantly enriched pathways between the URGs of the RNA-Seq data and the URPs of the TMT-MS data, there were 9 common pathways (Figure 6D and Supplemental Figure 14). Among them, while 6 terms were also significantly enriched in our existing high-throughput RNA-Seq data set using KLF4-transduced cells (5), “PPAR signaling pathway,” “Arginine and proline metabolism,” and “Toxoplasmosis” were not enriched, suggesting that these terms might be related to regulatory mechanisms of mocetinostat that are independent of upregulation of KLF4 (Supplemental Table 13).

### PGC-1α–dependent regulation of anabolic and catabolic genes by mocetinostat.

Among the 3 enriched terms specific to mocetinostat-treated cells, we focused on peroxisome proliferator-activated receptor (PPAR) pathway which mediates protective functions in cartilage and OA (Supplemental Table 13) (19). Analyzing expression changes of PPARs and PPARG coactivators by mocetinostat treatment in the high-throughput RNA-Seq dataset, only *PPARGC1A* was significantly upregulated (Supplemental Table 14). The *PPARGC1A* gene encodes PPAR γ coactivator 1-α (PGC-1α), which interacts with PPARG and is also a master regulator of mitochondrial biogenesis (20–22). Quantitative PCR (qPCR) also showed an approximately 30-fold increase of *PPARGC1A* expression by mocetinostat treatment in TC28a2 cells (Supplemental Figure 15). Furthermore, rates of PGC-1α^+^ cells were significantly increased in cartilage from mice that received mocetinostat injections (Supplemental Figure 16).

**Figure 7:**
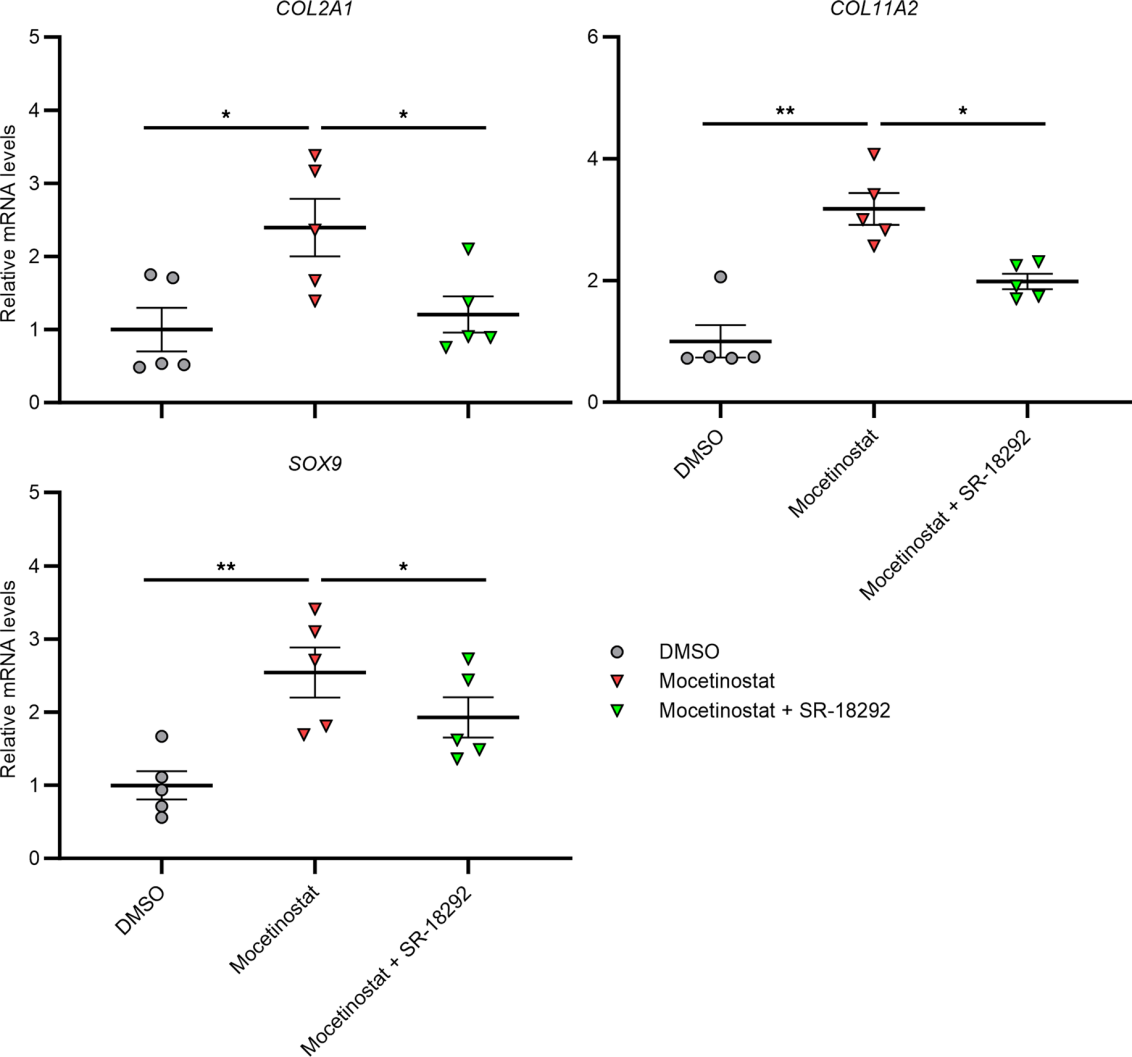
Chemical inhibition of PGC-1α in mocetinostat-treated OA chondrocytes. Cells were treated with 30 μM mocetinostat or DMSO (*n* = 5 donors). To inhibit PGC-1α activity, 50 μM of SR-18292 was used. RNA was collected 24 hours after initiation of treatment. mRNA levels are expressed as mean ± SEM, relative to DMSO. *P < 0.05, **P < 0.01, Dunnett’s test versus mocetinostat. Results of 1-way mixed-effects ANOVA test are shown in Supplemental Table 17.

We examined whether regulation of anabolic and catabolic genes by mocetinostat in human chondrocytes would be dependent on *KLF4* and *PPARGC1A*, using small interfering RNAs (siRNAs). While a knock-down efficiency of siKLF4 was about 92%, that of siPPARGC1A was about 67% (Supplemental Figure 17). Expression of cartilage signature genes, including *COL2A1*, *COL11A2*, and *SOX9*, was not affected by siRNA treatment against *KLF4* (Supplemental Figure 17). By siPPARGC1A treatment, upregulation of *SOX9* was significantly diminished, but expression levels of *COL2A1* and *COL11A2* did not change (Supplemental Figure 17). Meanwhile, siRNA-mediated knockdown of *PPARGC1A* significantly diminished suppression of catabolic and inflammatory genes, such as *PTGS2*, *MMP13*, and *ATAMTS5*, in IL-1β–stimulated chondrocytes (Supplemental Figure 18). Knockdown of *KLF4* did not have significant effects on expression of these genes (Supplemental Figure 18). We considered a possibility that expression of anabolic genes was not affected by siRNA-mediated knockdown of *PPARGC1A* because its knockdown efficiency was not sufficient. Therefore, we tested SR-18292, a PGC-1α inhibitor, in mocetinostat-treated chondrocytes. Treatment with SR-18292 diminished or eliminated upregulation of *COL2A1*, *COL11A2*, and *SOX9* by mocetinostat (Figure 7). Collectively, these findings demonstrate that mocetinostat upregulated anabolic genes and downregulated catabolic and inflammatory genes in chondrocytes, dependently on PGC-1α.

## Discussion

In this study, HTS for small molecules activating endogenous KLF4 expression identified mocetinostat, a class I selective HDAC inhibitor, as a potential DMOAD. In human joint tissue cells and BMSCs, mocetinostat showed chondrogenic and anabolic effects, and it suppressed hypertrophic, inflammatory, and catabolic genes, suggesting its promising profiles as a DMOAD. Furthermore, i.p. injections of mocetinostat in the mouse DMM model of experimental OA ameliorated pain behaviors and alleviated the severity of OA histopathological changes in cartilage, meniscus, synovium, and subchondral bone. Mechanistically, we showed that regulation of anabolic and catabolic genes by mocetinostat was dependent on PGC-1α.

HDAC enzymes modulate chromatin remodeling and gene expression profiles by removing acetyl groups from histones and other protein regulatory factors (23). HDACs are divided into 4 groups: class I (HDAC1, HDAC2, HDAC3, and HDAC8), class II (HDAC4, HDAC5, HDAC6, HDAC7, HDAC9, and HDAC10), class III (Sirtuins 1–7), and Class IV (HDAC11) (23, 24). Mocetinostat specifically binds to HDAC1, HDAC2, and HDAC3 and is classified as a class I selective HDAC inhibitor (25). Entinostat and chidamide, the other HDAC inhibitors that were hits in HTS and confirmed in a secondary screen, also belong to class I selective HDAC inhibitors (26, 27). These results obtained in our drug screening indicate that inhibition of class I HDACs upregulates expression of KLF4 (28–30). In a prior HTS, we searched for compounds activating expression of FOXO1 (31). FOXO1 is a transcription factor that regulates cellular aging and has protective functions in OA pathogenesis (17). Several HDAC inhibitors were identified as hit compounds in the HTS, and panobinostat, a pan-HDAC inhibitor, had the most desirable spectrum of activities in vitro and in vivo (31). Because *FOXO1* expression is regulated by KLF4 (5) and because the present study shows that mocetinostat upregulates *FOXO1* as well as *KLF4*, these 2 studies establish HDAC inhibitors as a group of potential DMOADs that target the 2 important transcription factors in joint tissues.

Expression of HDAC1 and HDAC2, members of class I HDACs, is increased in chondrocytes from patients with OA (32, 33), and diverse pathways and genes are associated with class I HDACs in regulation of OA pathogenesis in cartilage and chondrocytes. Snail family transcriptional repressor 1 interacts with HDAC1 and HDAC2 in repressing COL2A1 expression (32). HDAC1 increases activity of leukemia/lymphoma-related factors and suppresses *COMP* transcription in C3H10T1/2 cells (34). Knockdown of Protein kinase ϵ increases MMP13 secretion and RUNX2 expression, while upregulating gene expression of *HDAC2* (35). Furthermore, inhibition of HDAC3 suppresses nuclear transportation of NF-κB (36). The present study indicates that upregulation of cartilage signature genes and downregulation of catabolic and inflammatory genes by class I HDAC inhibition are associated with PGC-1α. Our findings that mocetinostat upregulates PGC-1α are consistent with a study reporting that inhibition of HDAC1 and HDAC2 increases expression of PGC-1α (21). PGC-1α is a master regulator of mitochondrial biogenesis and a transcription coactivator involved in a broad range of biological activities including skeletal homeostasis (20, 22, 37). PGC-1α limits oxidative stress and inhibits NF-κB signaling in OA pathogenesis (38, 39). PGC-1α is downregulated in OA-affected cartilage, and dysfunction of mitochondrial biogenesis promotes catabolic responses and reduces expression of cartilage ECM genes (38, 40, 41).

KLF4 directly regulates a variety of cartilage signature genes; furthermore, its effects on cartilage ECM genes are dependent on the PKA-RAP1-MEK-CREB axis (5). Meanwhile, KLF4 downregulates mediators of inflammation and ECM-degrading enzymes in human joint tissue cells via suppression of NF-κB activity (5, 42, 43). In the experiments shown in the present study, mocetinostat replicates these regenerative and protective effects of KLF4 in human joint tissue cells and BMSCs. Moreover, mocetinostat has antihypertrophic properties, suggesting an optimal profile as a DMOAD. Since our RNA-Seq analysis shows that interaction of mocetinostat with the PGC-1α pathway is independent from regulation of KLF4, mocetinostat would have the additional activities that are beneficial in OA pathogenesis. Expression of both KLF4 and PGC-1α can be regulated by mocetinostat through modulation of acetylation levels in their promoter regions, since inhibition or knockdown of HDACs increases histone acetylation in the promoter regions of *KLF4* and *PGC1A* genes (21, 28, 29).

Mocetinostat is undergoing clinical trials for various forms of cancers (44–47). I.p. injection of 20 mg/kg/day of mocetinostat in a congestive heart failure model of rats improved cardiac function without apparent systemic toxicity (48). Therefore, the doses of mocetinostat used in the present study — 2 and 10 mg/kg/day — will not be excessive for systemic administration in rodents. While there has been no study examining therapeutic effects of systemic administration of class I selective HDAC inhibitors in a mouse OA model, pan-HDAC inhibitors trichostatin A and panobinostat ameliorate the severity of OA in a surgically induced OA model of mice (31, 49). Pan-HDAC inhibitors have been applied in the treatment of a variety of diseases, especially cancer. However, large efforts have been made in clinical trials of more selective inhibitors of HDACs to reduce toxicity associated with broad inhibition of HDACs (50–52). The present study shows the potential of the class I selective HDAC inhibitor mocetinostat as a DMOAD, which may provide a therapeutic option with milder side-effects than nonselective inhibition of HDACs. However, it must be examined which types of HDAC inhibitors will be more therapeutically efficacious for OA. There are concerns of adverse effects for systemic and long-term administration of HDAC inhibitors to treat OA, including myelosuppression, diarrhea, cardiac effects, and osteoporosis (24, 53, 54). Development of sustained release formulations of mocetinostat for intraarticular administration would provide a feasible alternative for clinical application. A limitation in the present study is that all mouse experiments were done with only male animals and that sex differences were not addressed.

In conclusion, this study does show that the class I HDAC inhibitor mocetinostat has therapeutic and protective activities in cell and animal models of OA, qualifying it as a potential candidate for a DMOAD.

## Methods

### Mice.

All mice used in the experiments were males on the C57BL/6J background strain, and littermate mice were randomly assigned to the groups. Sample sizes were decided based on previous experience. There were no mice excluded from the analysis.

### Processing of human tissues and primary cell culture.

For human OA chondrocyte isolation, cartilage was harvested from tissues removed during knee-replacement surgery of patients with OA. For isolation of cells from normal human knees, meniscus and synovium were harvested from intact knee joints obtained from tissue banks within 48 hours postmortem. The donors with no history of joint disease or trauma were included, and all cartilage and meniscus surfaces were intact upon macroscopic inspection. Harvested tissues were digested with 2 mg/mL of type 2 collagenase overnight at 37°C. Isolated cells were maintained in DMEM with 10% calf serum (CS) supplemented with 1% penicillin-streptomycin-glutamine (PSG).

### Cell culture.

All cells were cultured at 37°C in a humidified atmosphere with 5% CO_2_. SW1353 cells were obtained from the American Type Culture Collection, and TC28a2 cells were purchased from Sigma-Aldrich. SW1353 cells, TC28a2 cells, and human primary cells were cultured in DMEM with 10% CS supplemented with 1% PSG. BMSCs were purchased from Lonza and were cultured in human Mesenchymal Stem Cell (hMSC) Growth BulletKit Medium (MSCGM) (Lonza). In pellet culture of BMSCs, hMSC Chondrogenic Differentiation BulletKit Medium (Lonza) was used. Human IL-1β (PeproTech) and TGF-β3 (PeproTech) were used to treat cells. The compounds used in screenings and validation experiments were provided by Calibr (a Division of Scripps Research Institute). Mocetinostat for other experiments was obtained from LC Laboratories, and SR-18292 was purchased from Selleck Chemicals. Stocks of the compounds were diluted in DMSO. Cell viability was measured with Countess II FL Automated Cell Counter (Thermo Fisher Scientific) using Trypan Blue stain 0.4% (Thermo Fisher Scientific).

### Establishment of the monoclonal KLF4 reporter cell line.

We inserted the HiBiT sequence (5′-GTGAGCGGCTGGCGGCTGTTCAAGAAGATTAGC-3′) into the site immediately after the first ATG codon of *KLF4* gene in SW1353 cells by electroporation with ribonucleoprotein complexes, which consisted of recombinant Cas9 nuclease and synthetic guide RNA (5′-UCUGGGCCCCCACAUUAAUG-3′) in the presence of a single-stranded oligodeoxynucleotide template (5′-GTGAGCGGCTGGCGGCTGTTCAAGAAGATTAGCGGAGGAGGTGGTTCTGGTGGTGGAGGTAGC-3′), as shown in Supplemental Figure 1. Expression of HiBiT in the edited cell pools was confirmed by measuring luminescence of cell lysates using Nano-Glo HiBiT Lytic Detection System (Promega), according to the manufacturer’s instruction. Successful integration of the HiBiT sequence at the intended loci was confirmed by Sanger sequencing. The monoclonal KLF4 reporter cells were established by serial limiting dilution.

### HTS with the ReFRAME library.

The ReFRAME library is composed of 11,948 small molecules that have reached clinical development or have undergone significant preclinical profiling (8, 9). The compounds were prespotted into 1,536-well assay plates with the Echo Acoustic Liquid Handler (Beckman Coulter) to achieve a final concentration of 5 μM in a final assay volume of 8 μL per well. The KLF4 reporter SW1353 cells were dispensed into the assay plates with 100 cells per well and were allowed to grow for 24 hours. Quantitation of luminescence activity was performed Nano-Glo HiBiT Lytic Detection System, and the raw signal intensities were normalized across each plate using *Z* scores. Compounds with *Z* scores above 2 SDs were replated in triplicate for confirmation assays and evaluated in 10-point dose-response concentration curves. EC_50_ for each compound was calculated from the dose-response curves, and EC_50_ < 1 μM was adopted as a criterion for the validated hit selection.

### RNA isolation and qPCR.

Total RNA was isolated with Direct-zol RNA MicroPrep kit (Zymo Research) and was reverse transcribed using PrimeScript RT Reagent kit (TaKaRa Bio). qPCR was performed on a LightCycler 96 instrument (Roche) using TaqMan probes (Thermo Fisher Scientific) listed in Supplemental Table 15. mRNA levels were normalized with *GAPDH*.

### DMM surgery and i.p. injections in mice.

The mouse surgical OA model was induced by DMM surgery (11). In brief, the surgical approach to the knee was the medial parapatellar. The fat pad was dissected, and the medial meniscus (MM) and the medial meniscotibial ligament (MMTL) were identified. The MMTL was transected, and the MM was confirmed to be freely displaceable. The operators were blinded to treatment groups.

I.p. injections of 2 mg/kg or 10 mg/kg of mocetinostat or vehicle were executed 3 times a week starting 1 week after DMM or Sham surgery. Mocetinostat was first diluted in DMSO and was further diluted in 5% dextrose at a concentration of 0.2 mg/mL for the 2 mg/kg group and 1 mg/mL for the 10 mg/kg group. The final concentration of DMSO was adjusted to 1.67% for all groups. Those who performed injections were aware of the group allocation. The mice were monitored for water and food intake and were inspected for activity and hair appearance 3 times per week. Knees were harvested at 10 weeks after surgery for histological evaluation.

### von Frey test.

To evaluate mechanical allodynia in mice, von Frey filaments with 5 different target forces (no. 1, 0.04 g; no. 2, 0.16 g; no. 3, 0.4 g; no. 4, 1 g; and no. 5, 2 g; Touch Test Sensory Evaluators; North Coast Medical) were used for mechanical stimulation to the plantar surface of the hind paw, as previously described (12, 13). Numbers of paw withdrawals from 5 stimulations per filament per mouse were counted. Mice were tested in randomized order at each time point.

### Histological analyses.

Mouse knee joint tissues were fixed in Z-fix (Anatech) for 2 days and were decalcified in TBD-2 (Thermo Fisher Scientific) for a further 2 days. The samples were embedded in paraffin and were sectioned in 4 μm thickness. Safranin-O and fast green staining was performed according to the standard protocols.

Histological scoring of OA for the medial femoral condyle and tibial plateau was performed using the summed OARSI scores (score, 0–48) (14). Meniscus histopathological scores were graded by Kwok’s meniscus scoring system (score, 0–50) (15). Synovial changes were evaluated using Krenn’s synovitis scoring system (score, 0–9) (16). All scorings were performed in a blinded fashion.

### IHC.

Sections were deparaffinized, washed, and blocked with 2.5% horse serum (Vector Laboratories) for 1 hour at room temperature. They were incubated with primary antibodies shown in Supplemental Table 16 overnight at 4°C. Goat or rabbit IgG (Vector Laboratories) was used as a negative control. Immune complexes were detected using VECTASTAIN Elite ABC-HRP Kits (Vector Laboratories). Sections were incubated with DAB and then were counterstained with hematoxylin and methyl green. For quantification of positive cells in IHC samples of mouse knees, calculation of positive cell rates was obtained from fields in the medial tibial plateau.

### High-throughput RNA-Seq analysis.

TC28a2 cells were treated with 2 μM of mocetinostat or DMSO for 24 hours, and total RNA was collected. RNA-Seq libraries were constructed using an early access High Throughput RNA-Seq Prep kit (Jumpcode Genomics). Briefly, 20 ng of total RNA per sample was fragmented by heat, followed by reverse transcription and ligation of adapter at the 3′ end of the cDNA. Libraries were PCR amplified to add full-length adapter sequences and index barcodes (i5 and i7). After PCR, libraries were treated with the CRISPRclean Bulk Ribodepletion Reagents (Human, Mouse, Rat) (Jumpcode Genomics) per manufacturer’s recommended protocol and were sequenced with single-read 100 bp on a NextSeq2000 instrument (Illumina) at an average of 5 million reads per sample.

The high-throughput RNA-Seq data were analyzed using nf-core/RNA-Seq pipeline version 1.4.2 (implemented on Nextflow version 20.07.1), which is an open-source and available at https://github.com/nf-core/rnaseq/commit/3b6df9bd104927298fcdf69e97cca7ff1f80527c as part of the nf-core project (55). Briefly, the reads were trimmed for adapters with trimGalore! version 0.6.4 (https://www.bioinformatics.babraham.ac.uk/projects/trim_galore/), and reads mapping to ribosomal RNA were removed using SortMeRNA version 2.1b (56). The remaining reads were aligned to the human reference genome (GRCh38 ENSEMBL build 98) with STAR version 2.6.1d (57). Gene-level assignment was performed using featureCounts version 1.6.4 (58). The gene expression matrix with raw gene counts was then used for differential gene expression analysis with the Bioconductor DESeq2 R package version 1.20.0 (59).

The resulting *P* values were adjusted using the Benjamini-Hochberg’s approach for controlling the FDRs. Genes with an FDR < 0.05 and a |log_2_(FC)| > 1 were considered significantly differentially expressed. DAVID Bioinformatics Resources (v2022q3; https://david.ncifcrf.gov/) was used for KEGG pathway analysis (18). Pathways satisfying an FDR <0.05 was considered significant.

### siRNA knockdown experiments.

siRNAs for KLF4 (s17794; Thermo Fisher Scientific), PPARGC1A (s21394; Thermo Fisher Scientific), and the negative control (4390843; Thermo Fisher Scientific) were used for knockdown experiments. Cells were transfected with siRNA using Lipofectamine RNAiMAX transfection reagent (Thermo Fisher Scientific), following manufacturer’s protocol.

### TMT-MS analysis.

TC28a2 cells were treated with 2 μM of mocetinostat or DMSO for 24 hours, and cells were lysed in Pierce RIPA Buffer supplemented with 1% Halt protease and phosphatase inhibitor cocktail (Thermo Fisher Scientific) to collect protein lysates. Total protein was quantified using Pierce Rapid Gold BCA Protein Assay Kit (Thermo Fisher Scientific). Protein lysates were precipitated with 25% trichloroacetic acid overnight. The dried precipitated protein pellet was resuspended in 50 mM triethylammonium bicarbonate (pH 8.5) containing 8M urea. The protein samples were alkylated with 5 mM tris (2-carboxyethyl) phosphine and were reduced with 10 mM chloroacetamide. Next, the samples were digested with trypsin overnight at 37°C and were desalted with C18 Spin Columns (Thermo Fisher Scientific). The desalted peptides were labeled with TMT isobaric tags (Thermo Fisher Scientific, lot XA338782) following the previously published protocol (60). The labeled peptides were combined into 1 tube and dried via a Speed-Vac, and the peptides were fractionated using High pH Reversed-Phase Peptide Fractionation Kit (Thermo Fisher Scientific).

The TMT-labeled samples were analyzed on an Orbitrap Fusion Lumos Tribrid Mass Spectrometer (Thermo Fisher Scientific). Samples were injected directly onto a 25 cm, 100 μm ID column packed with BEH 1.7 μm C18 resin (Waters) and were separated at a flow rate of 300 nL/minute on an Easy-nLC 1200 System (Thermo Fisher Scientific). Buffers A and B were 0.1% formic acid in water and 90% acetonitrile, respectively. A gradient of 1%–25% B over 120 minutes, an increase to 40% B over 40 minutes, an increase to 100% B over 10 minutes, and a hold at 100% B for 10 minutes was used for a 180-minute total run time. Peptides were eluted directly from the tip of the column and nanosprayed directly into the mass spectrometer by application of 2.5 kV voltage at the back of the column. The Lumos was operated in a data-dependent mode. Full MS1 scans were collected in the Orbitrap at 120,000 resolution. The cycle time was set to 3 seconds, and within these 3 seconds, the most abundant ions per scan were selected for collision-induced dissociation MS2 in the ion trap. MS3 analysis with multinotch isolation was utilized for detection of TMT reporter ions at 60,000 resolution. Monoisotopic precursor selection was enabled, and dynamic exclusion was used with an exclusion duration of 10 seconds.

### MS data analysis.

The MS spectra were analyzed by Proteome Discoverer 2.5 (Thermo Fisher Scientific) and were searched using the Uniprot human protein database including protein isoforms (version 2022-08-03) and a list of common protein contaminants. The decoy database was the reverse of this Uniprot database to filter identifications to an FDR of 0.01. The processing workflow used was Tribid_TMT_Quan_SPS_MS3_SequestHT_Percolator, and the consensus workflow was Comprehensive_EnhancedAnnotation_Reporter_Quan. Parameters were specified as follows: peptide length between 7 and 45 amino acids, fully tryptic or semitryptic digestion, and a maximum of 2 miscleavages. The static modification searched for were TMT tags on lysine residues, peptide N-termini (+229.162932 Da), and carbamidomethylation of cysteine residues (+57.021464 Da). Reporter ion distributions specific to the lot number of the TMT reagent were employed as correction factors.

Statistical analysis and fold changes were calculated by Proteome Discoverer 2.5. The resulting *P* values were adjusted using the Benjamini-Hochberg approach for controlling the FDRs. Proteins with an FDR < 0.05 were considered to be significantly differentially expressed proteins. DAVID Bioinformatics Resources (v2022q3) was used for KEGG pathway analysis. Pathways satisfying an FDR < 0.05 was considered significant.

### Statistics.

Results were analyzed using GraphPad Prism version 9.4.0 (GraphPad Software). Two-tailed paired *t* test was used to establish statistical significance between the 2 groups. One-way mixed-effects ANOVA followed by Dunnett’s test, 2-way mixed-effects ANOVA followed by Sidak’s multiple comparison test, and Kruskal-Wallis followed by Dunn’s test were used for multiple-group comparisons. Results of omnibus tests for multiple comparisons are shown in Supplemental Table 17, and other statistical tests used are all described in each figure legend. In statistical analyses of qPCR data, log_2_ transformed values were used to assume normal distribution. Biologically independent sample numbers were shown in legends. For micrographs, representative images in each group were displayed.

### Study approval.

All human tissues were obtained with approval by the Scripps Human Subjects Committee (IRB no. 15-6648). All animal studies were performed with approval by the Scripps IACUC (no. 09-0130-05). We complied with all relevant ethical regulations.

### Data availability.

The raw data for high-throughput RNA-Seq analyses are deposited in the Gene Expression Omnibus (www.ncbi.nlm.nih.gov/geo/) under accession nos. GSE220755 and GSE183956. Raw data are available in the Supporting Data Values file and from the corresponding author upon request.

## Author contributions

MKL, KAJ, and JRY designed the study. MK, DBM, JKD, and MO performed the experiments. MK, DBM, JKD, and MO analyzed the data. MK and MKL drafted the paper, which was approved by all coauthors.

## Supplementary Material

Supplemental data

Supplemental table 17

Supplemental tables 5-12

Supporting data values

## Version 1 09/08/2023

Electronic publication
