# Supplementary material for: Mocetinostat activates Krüppel-like factor 4 and protects against tissue destruction and inflammation in osteoarthritis
Source: JCI Insight. 2023 Sep 8;8(17):e170513. doi: 10.1172/jci.insight.170513 (PMC10544226; doi:10.1172/jci.insight.170513)
Supplement: Supplemental data [file jciinsight-8-170513-s242.pdf]

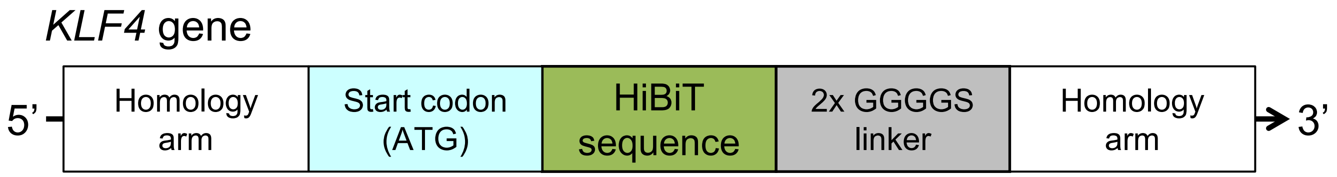

Supplemental Figure 1. A schema for *KLF4* gene editing in SW1353 cells to establish the KLF4 reporter cell line.

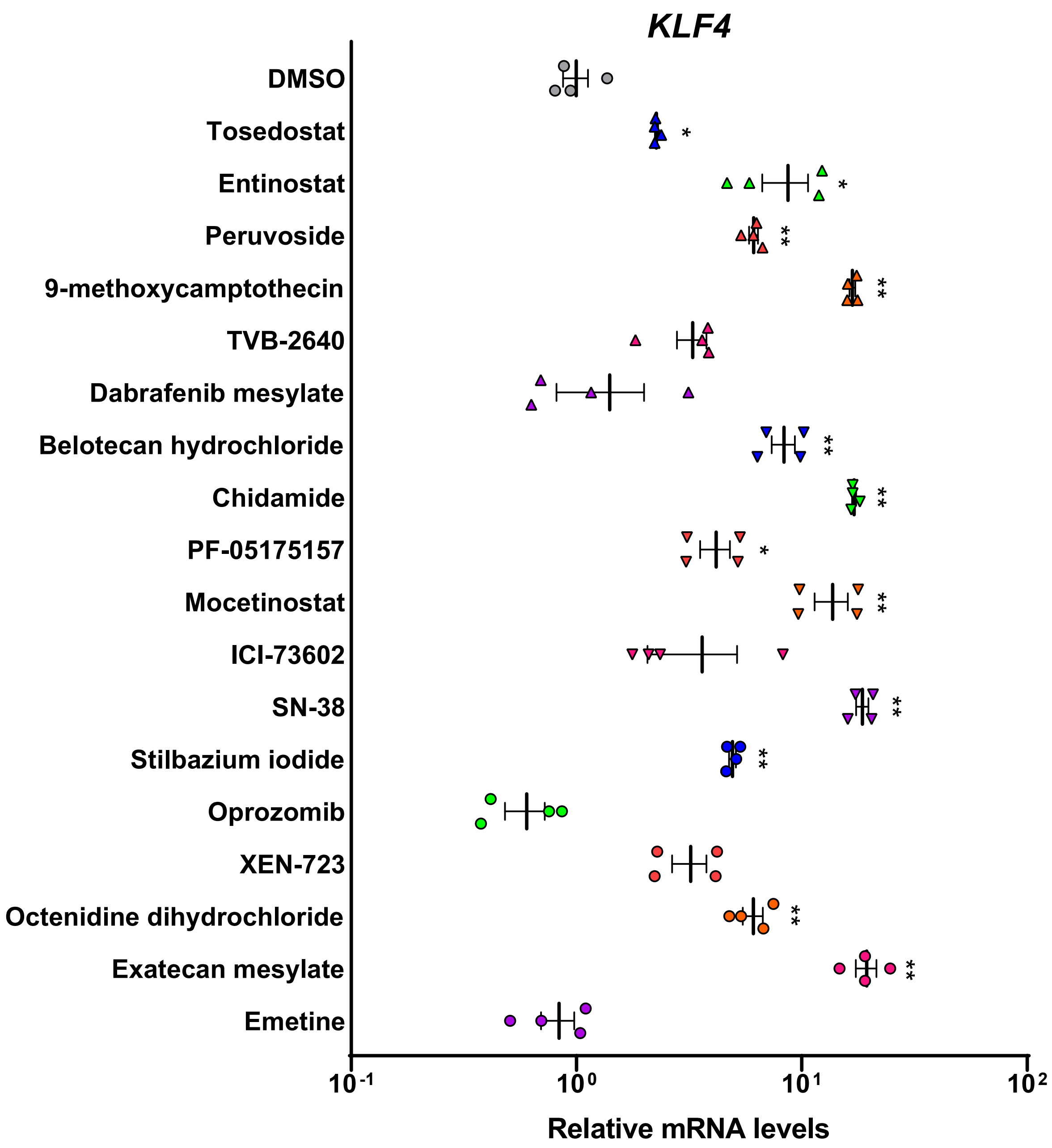

Supplemental Figure 2. ***KLF4*** **expression levels in SW1353 cells treated with the identified compounds.** Cells were treated with the indicated compounds at the tolerated doses or DMSO, and RNA was collected 24 hours after initiation of treatment. mRNA levels are expressed as means±SE, relative to DMSO (n=4 from four independent experiments). *P<0.05, **P<0.01, Dunnett's test versus DMSO. Results of one-way mixed-effects ANOVA test are shown in Supplemental Table 17.

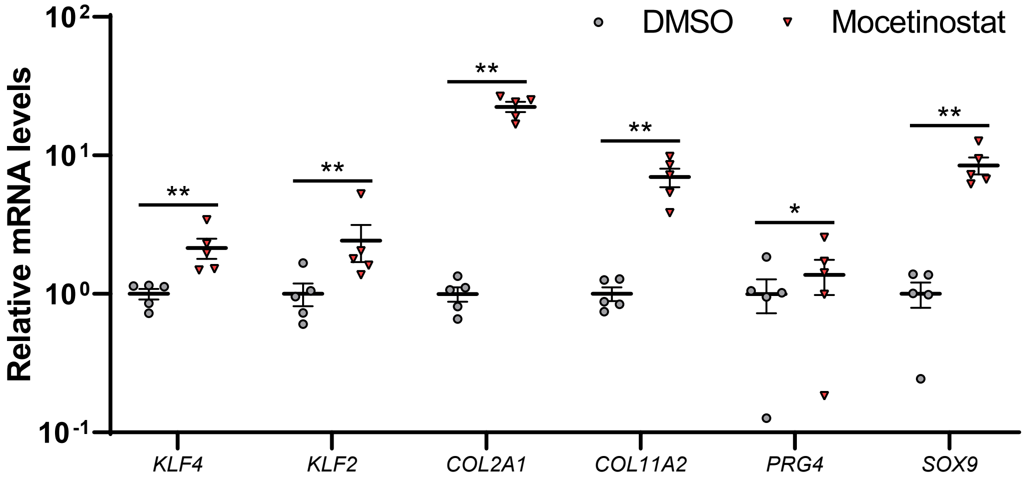

Supplemental Figure 3. Regulation of chondrogenic genes by mocetinostat in human BMSCs during monolayer culture. Cells were treated with 10 μM of mocetinostat or DMSO, and RNA was collected 24 hours after initiation of treatment. mRNA levels are expressed as means±SE, relative to DMSO (n=5 donors). *P<0.05, **P<0.01, paired t-test.

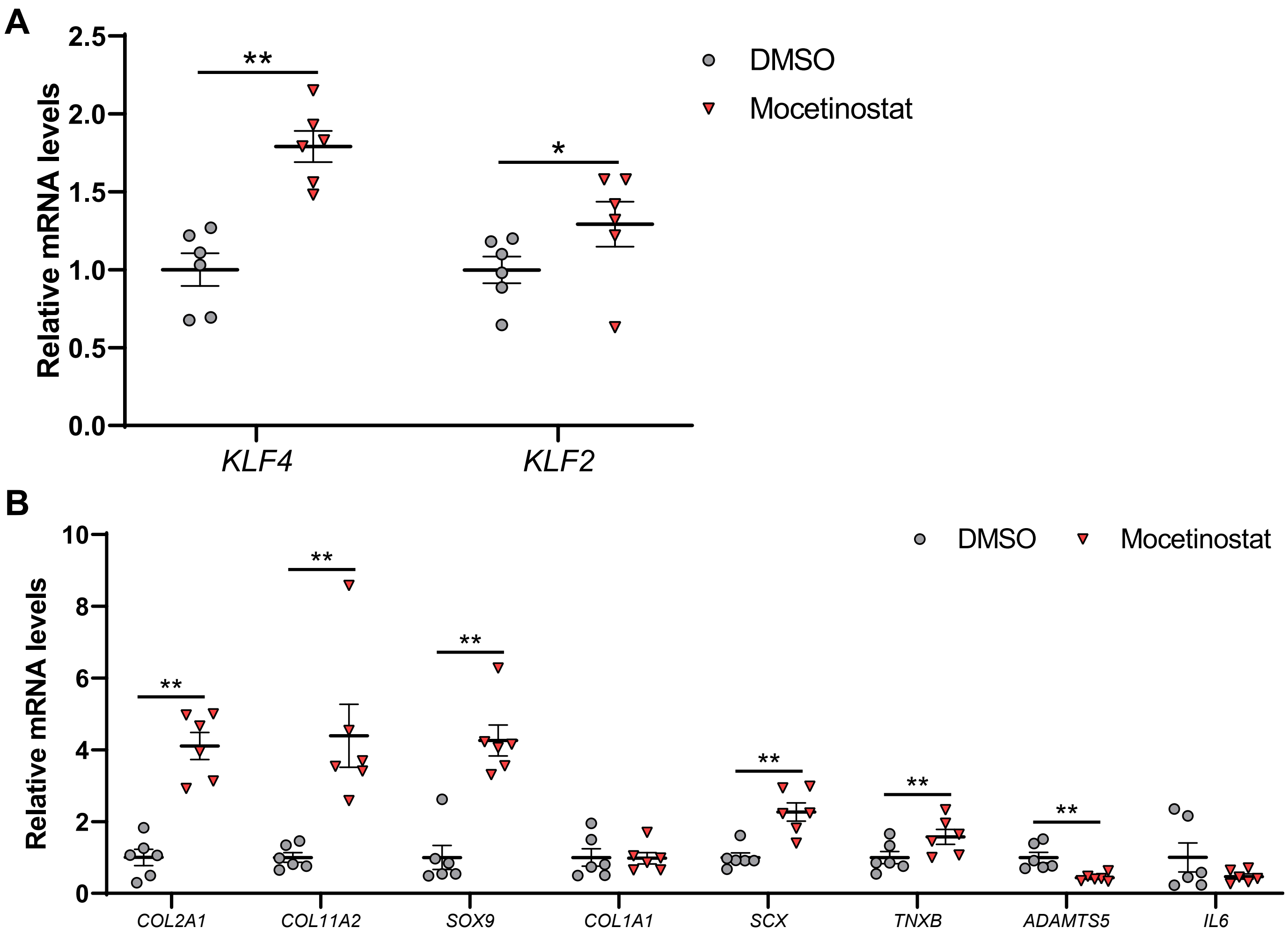

Supplemental Figure 4. Regulation of anabolic and catabolic genes by mocetinostat in human meniscal cells. (**A**, **B**) Cells were treated with 10 μM of mocetinostat or DMSO, and RNA was collected 24 hours after initiation of treatment. mRNA levels are expressed as means±SE, relative to DMSO (n=6 donors). *P<0.05, **P<0.01, paired t-test.

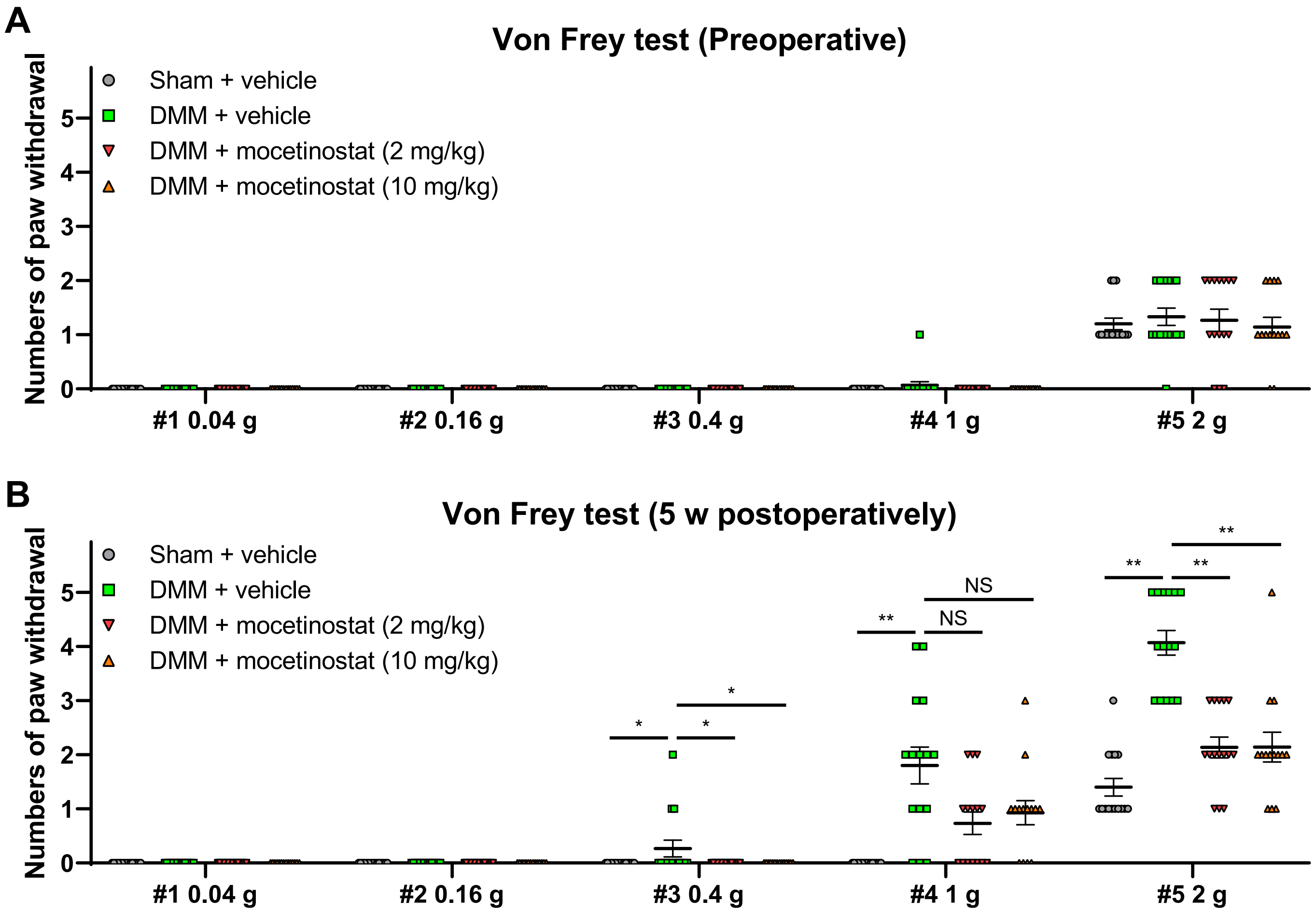

Supplemental Figure 5. Von Frey test in mocetinostat-treated mice after DMM surgery. (**A**, **B**) Related to the experiments in Figure 5, results of von Frey test preoperatively (A) and at 5 weeks postoperatively (B) in mocetinostat-treated mice after DMM surgery are shown. Numbers of paw withdrawal from 5 stimulations per filament per mouse are expressed as means±SE. n=14 for DMM + 10 mg/kg of mocetinostat, and n=15 for the other groups. *P<0.05, **P<0.01, Dunn's test versus DMM + vehicle. NS, not significant. Results of Kruskal-Wallis test are shown in Supplemental Table 17.

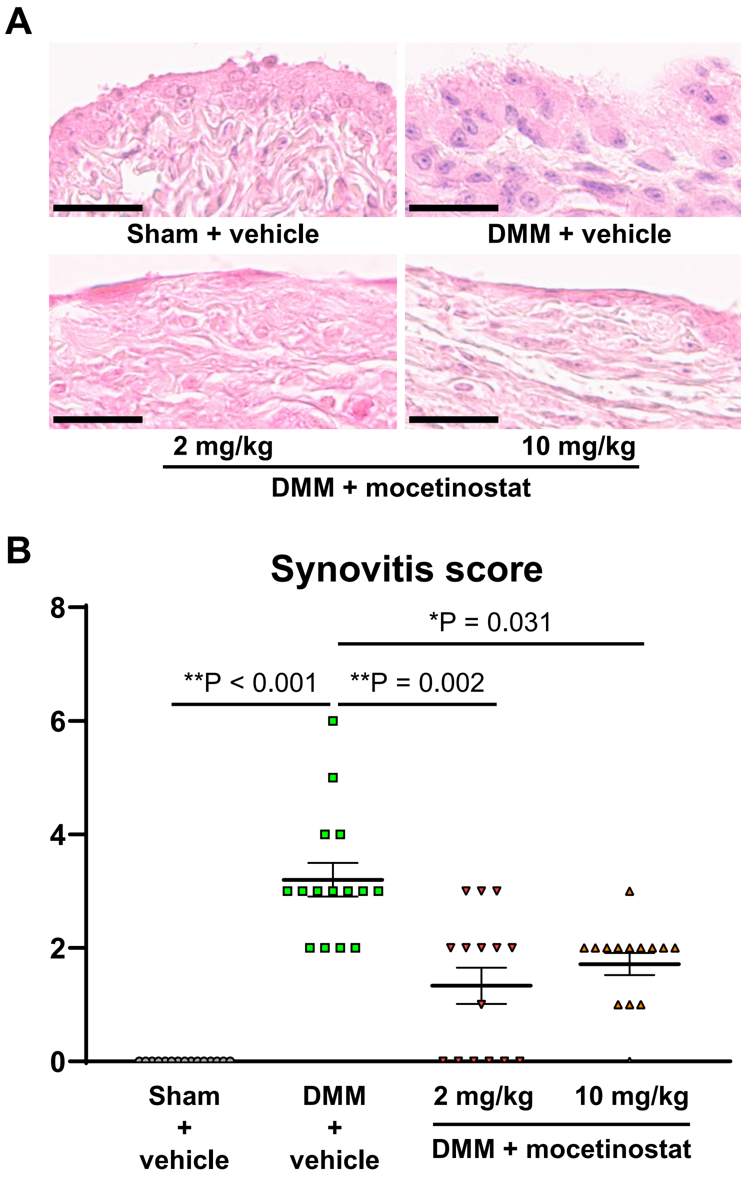

Supplemental Figure 6. Synovitis scores in mocetinostat-treated mice after DMM surgery. This figure shows data related to the experiments in Figure 5. n=14 for DMM + 10 mg/kg of mocetinostat, and n=15 for the other groups. (**A**) Representative HE staining images of synovium for each group. Scale bars, 15 µm. (**B**) Synovitis scores. *P<0.05, **P<0.01, Dunn's test versus DMM + vehicle. All quantitative data are expressed as means±SE, and results of Kruskal-Wallis test are shown in Supplemental Table 17.

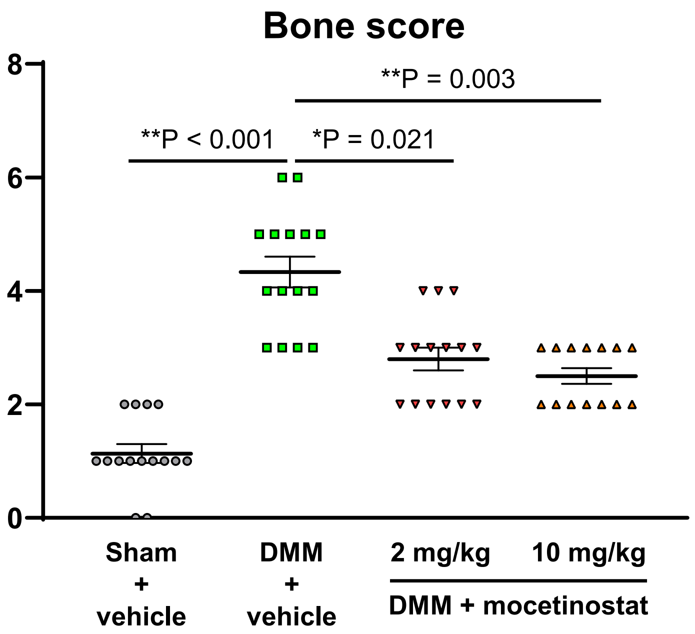

Supplemental Figure 7. Bone scores in mocetinostat-treated mice after DMM surgery. This figure shows data related to the experiments in Figure 5. n=14 for DMM + 10 mg/kg of mocetinostat, and n=15 for the other groups. *P<0.05, **P<0.01, Dunn's test versus DMM + vehicle. All quantitative data are expressed as means±SE, and results of Kruskal-Wallis test are shown in Supplemental Table 17.

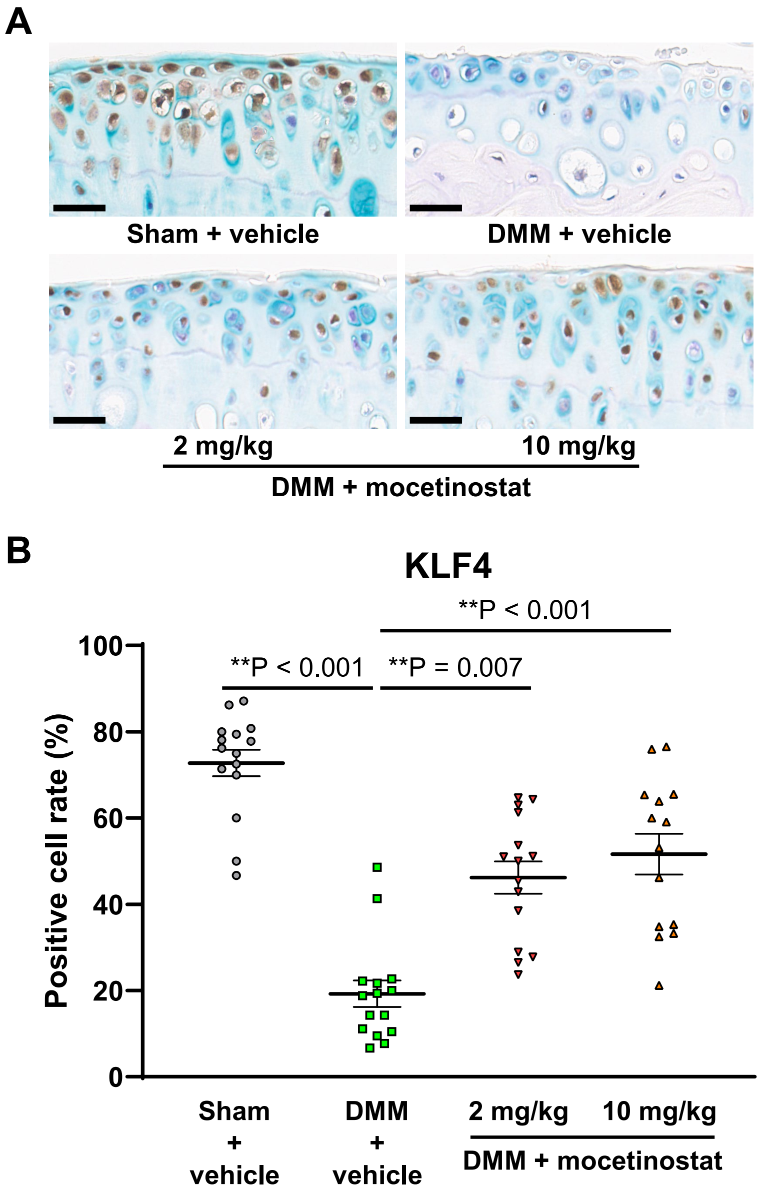

Supplemental Figure 8. KLF4 expression in mocetinostat-treated mice after DMM surgery. This figure shows data related to the experiments in Figure 5. (**A**, **B**) Immunohistochemistry for KLF4 in knee cartilage was performed. n=14 for DMM + 10 mg/kg of mocetinostat, and n=15 for the other groups. Scale bars, 60 μm. *P<0.05, **P<0.01, Dunn's test versus DMM + vehicle. All quantitative data are expressed as means±SE, and results of Kruskal-Wallis test are shown in Supplemental Table 17.

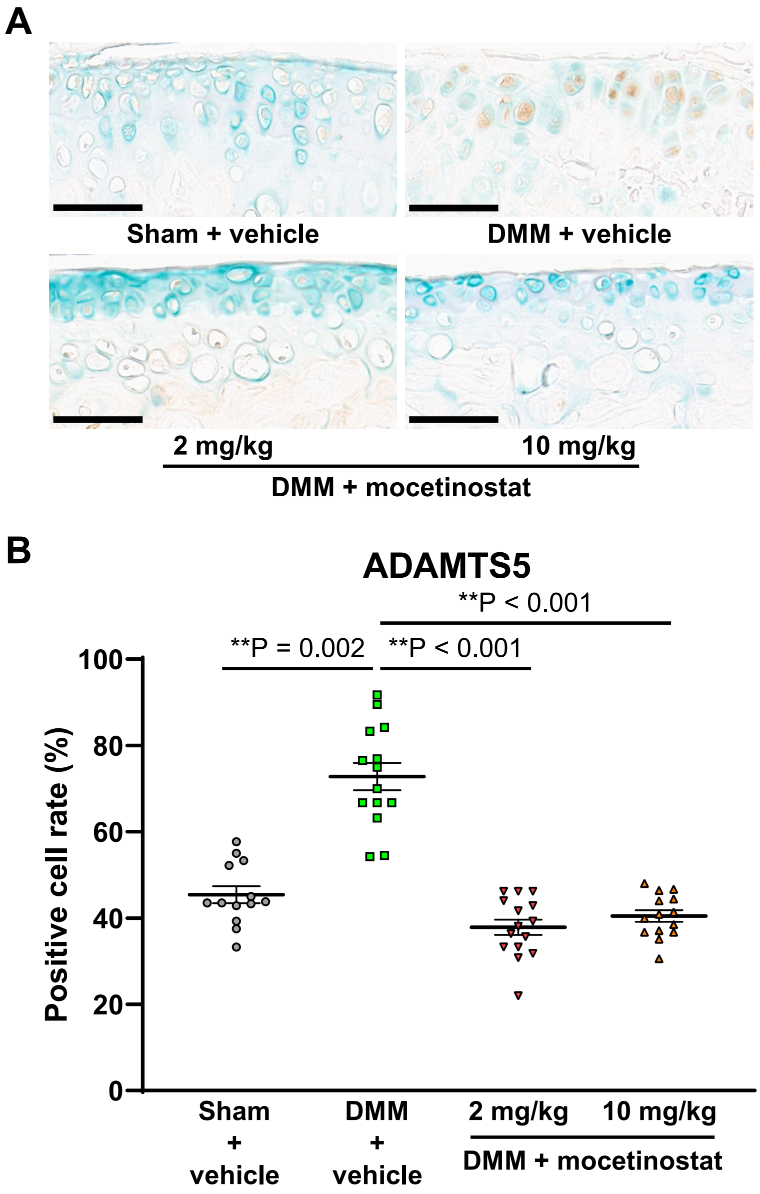

Supplemental Figure 9. ADAMTS5 expression in mocetinostat-treated mice after DMM surgery. This figure shows data related to the experiments in Figure 5. (**A**, **B**) Immunohistochemistry for ADAMTS5 in knee cartilage was performed. n=13 for Sham + vehicle, n=14 for DMM + vehicle, n=15 for DMM + 2 mg/kg of mocetinostat, and n=14 for DMM + 10 mg/kg of mocetinostat. Scale bars, 60 µm. *P<0.05, **P<0.01, Dunn's test versus DMM + vehicle. All quantitative data are expressed as means±SE, and results of Kruskal-Wallis test are shown in Supplemental Table 17.

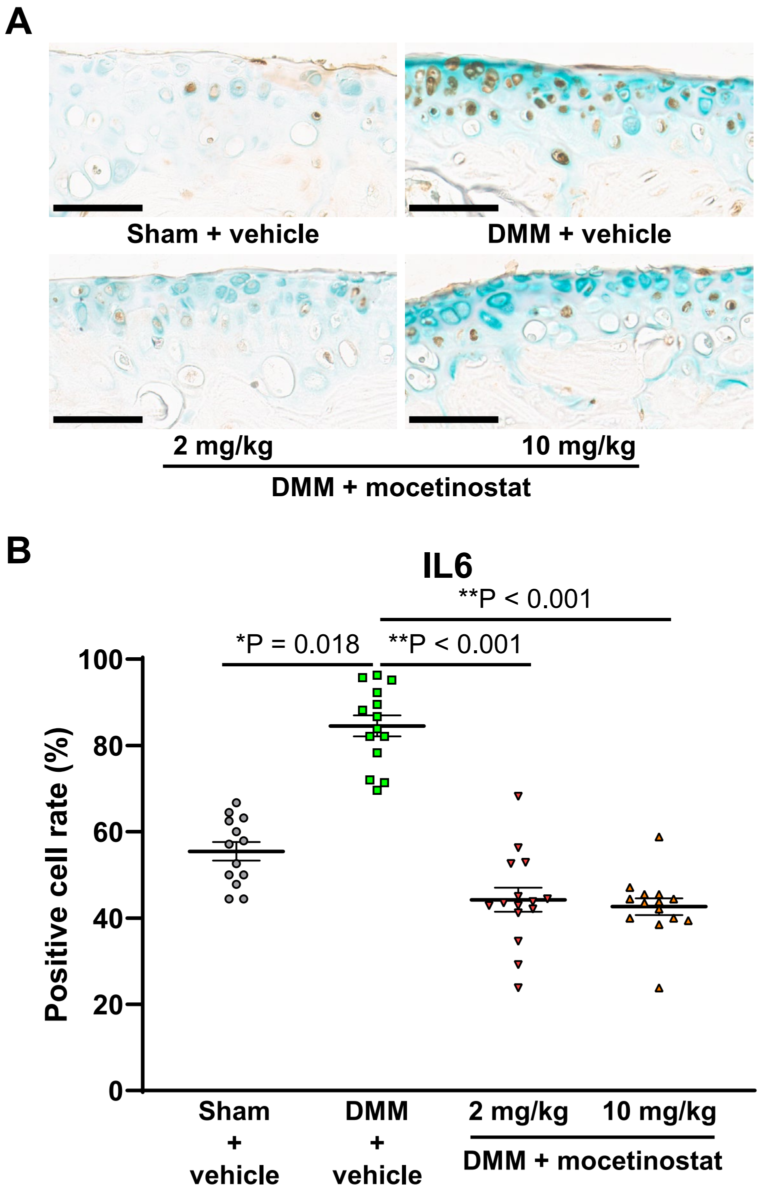

Supplemental Figure 10. IL6 expression in mocetinostat-treated mice after DMM surgery. This figure shows data related to the experiments in Figure 5. (**A**, **B**) Immunohistochemistry for IL6 in knee cartilage was performed. n=13 for Sham + vehicle, n=14 for DMM + vehicle, n=15 for DMM + 2 mg/kg of mocetinostat, and n=14 for DMM + 10 mg/kg of mocetinostat. Scale bars, 60 µm. *P<0.05, **P<0.01, Dunn's test versus DMM + vehicle. All quantitative data are expressed as means±SE, and results of Kruskal-Wallis test are shown in Supplemental Table 17.

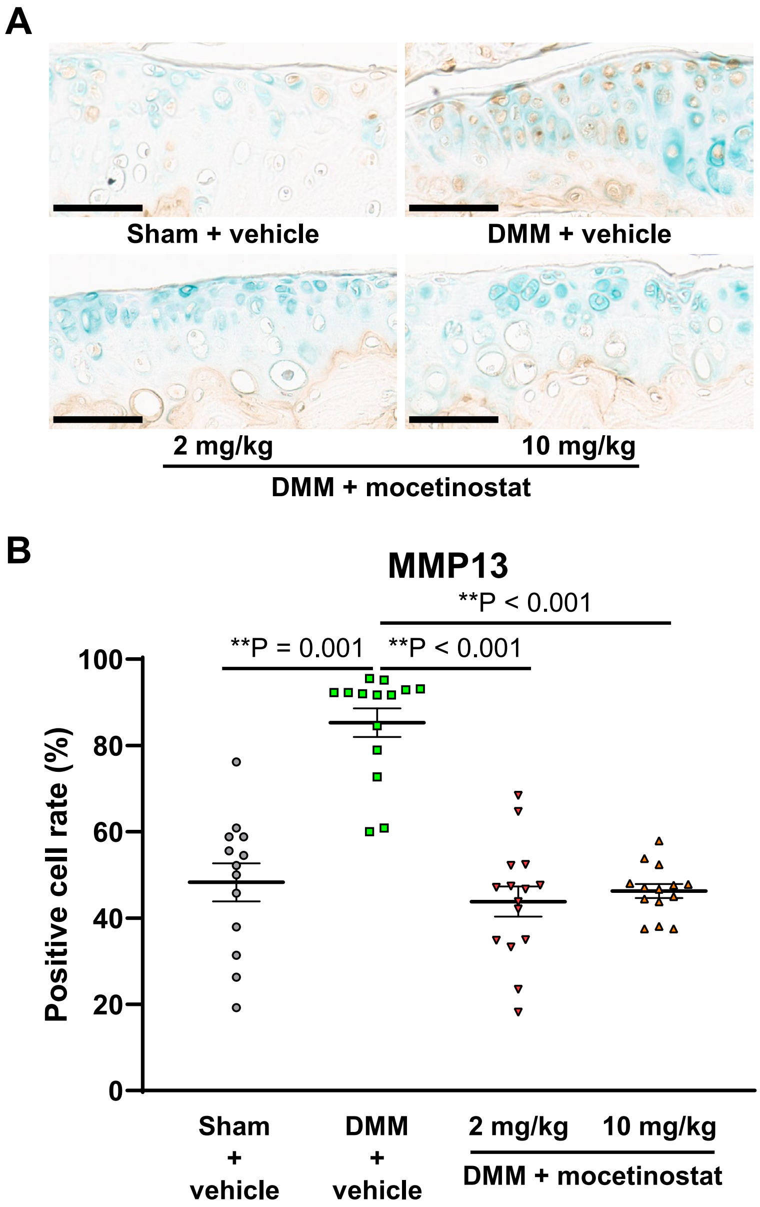

Supplemental Figure 11. MMP13 expression in mocetinostat-treated mice after DMM surgery. This figure shows data related to the experiments in Figure 5. (**A**, **B**) Immunohistochemistry for MMP13 in knee cartilage was performed. n=13 for Sham + vehicle, n=14 for DMM + vehicle, n=15 for DMM + 2 mg/kg of mocetinostat, and n=14 for DMM + 10 mg/kg of mocetinostat. Scale bars, 60 µm. *P<0.05, **P<0.01, Dunn's test versus DMM + vehicle. All quantitative data are expressed as means±SE, and results of Kruskal-Wallis test are shown in Supplemental Table 17.

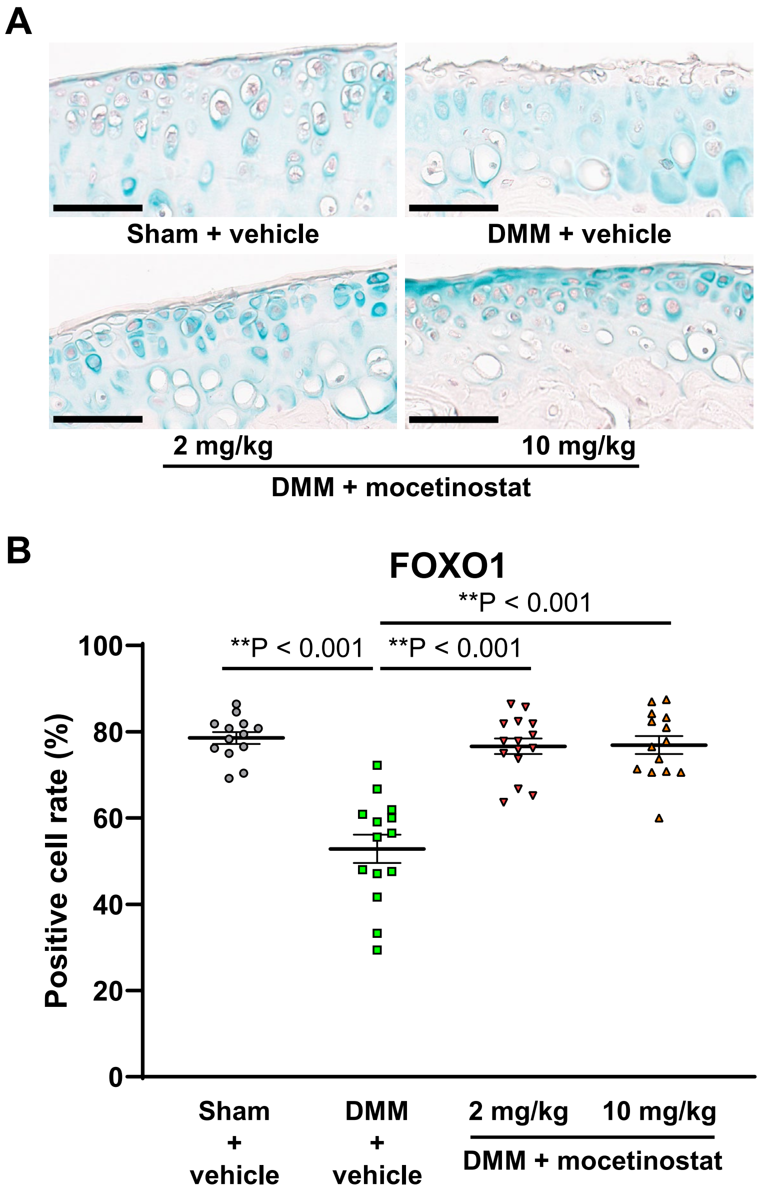

Supplemental Figure 12. FOXO1 expression in mocetinostat-treated mice after DMM surgery. This figure shows data related to the experiments in Figure 5. (**A**, **B**) Immunohistochemistry for FOXO1 in knee cartilage was performed. n=13 for Sham + vehicle, n=14 for DMM + vehicle, n=15 for DMM + 2 mg/kg of mocetinostat, and n=14 for DMM + 10 mg/kg of mocetinostat. Scale bars, 60 µm. *P<0.05, **P<0.01, Dunn's test versus DMM + vehicle. All quantitative data are expressed as means±SE, and results of Kruskal-Wallis test are shown in Supplemental Table 17.

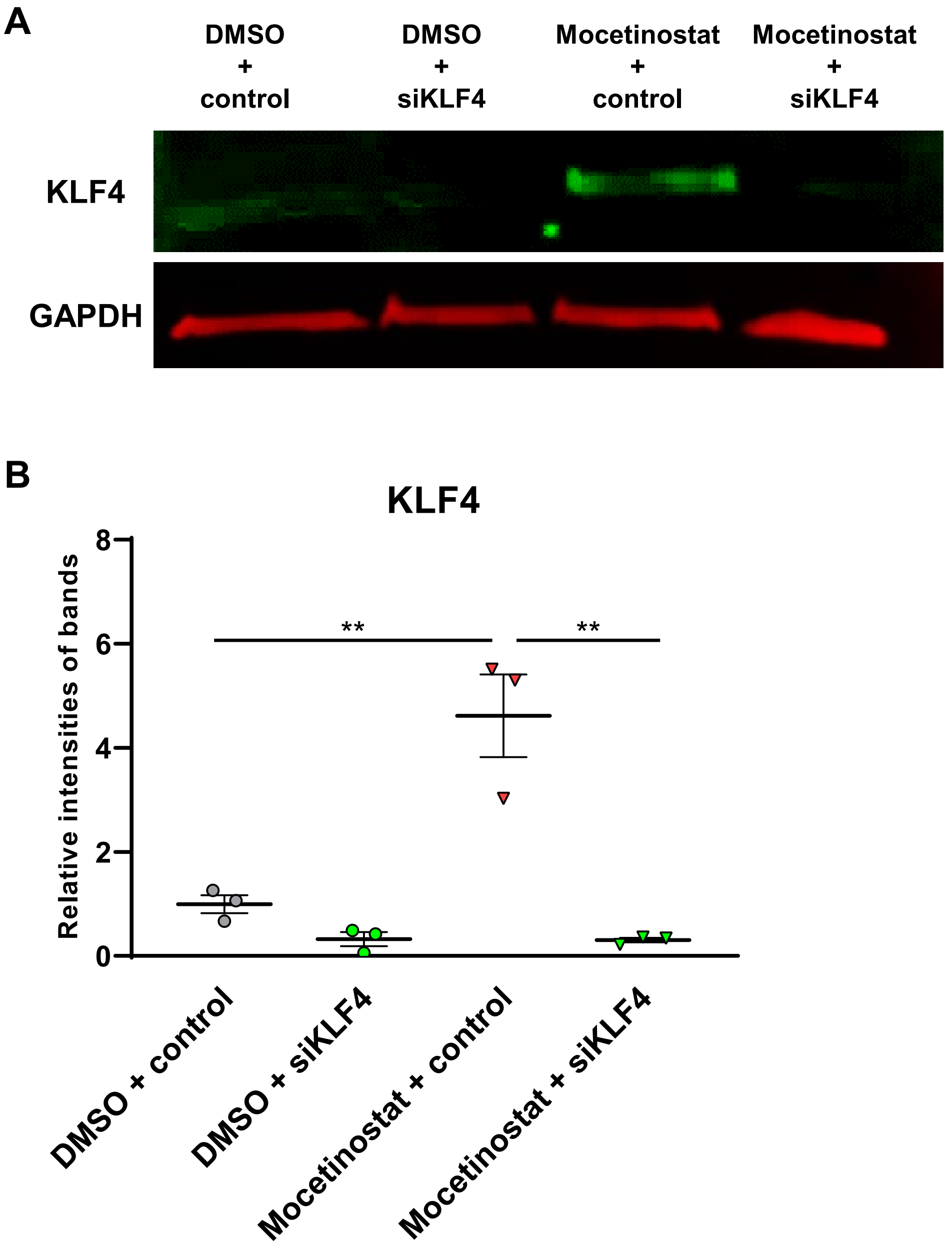

Supplemental Figure 13. Western blotting analysis of mocetinostat-treated TC28 cells. Cells were transfected with siRNAs, and were treated with 2 μM mocetinostat or DMSO. Total protein was collected 24 hours after initiation of treatment. (**A**) Representative blots from three independent experiments. (**B**) Normalized intensities of protein bands are expressed as means±SE, relative to DMSO + control (n=3 independent experiments). *P<0.05, **P<0.01, Sidak's multiple comparison test. Results of two-way mixed-effects ANOVA test are shown in Supplemental Table 17.

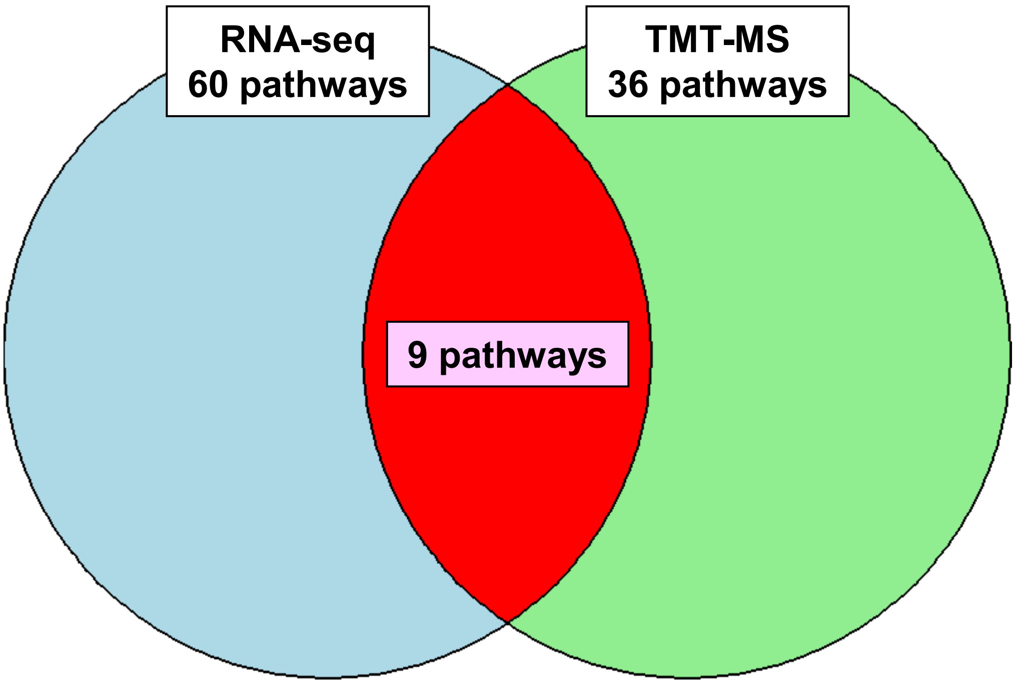

Supplemental Figure 14. Venn diagram of enriched pathways in KEGG pathway analysis for the URGs of the RNA-seq data and the URPs of the TMT-MS data.

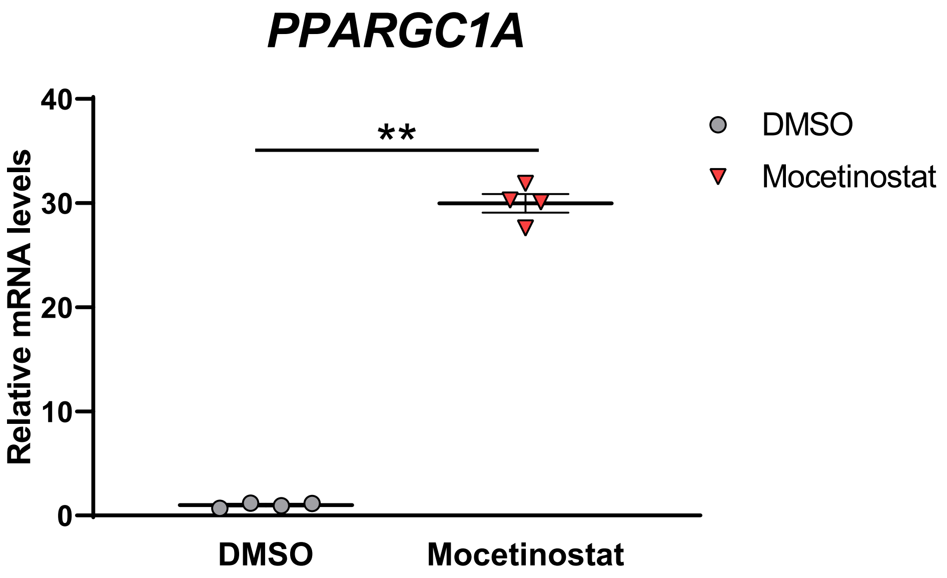

Supplemental Figure 15. Regulation of *PPARGC1A* by mocetinostat in TC28a2 cells. Cells were treated with 2 μM of mocetinostat or DMSO, and RNA was collected 24 hours after initiation of treatment. mRNA levels are expressed as means±SE, relative to DMSO (n=4 independent experiments). *P<0.05, **P<0.01, paired t-test.

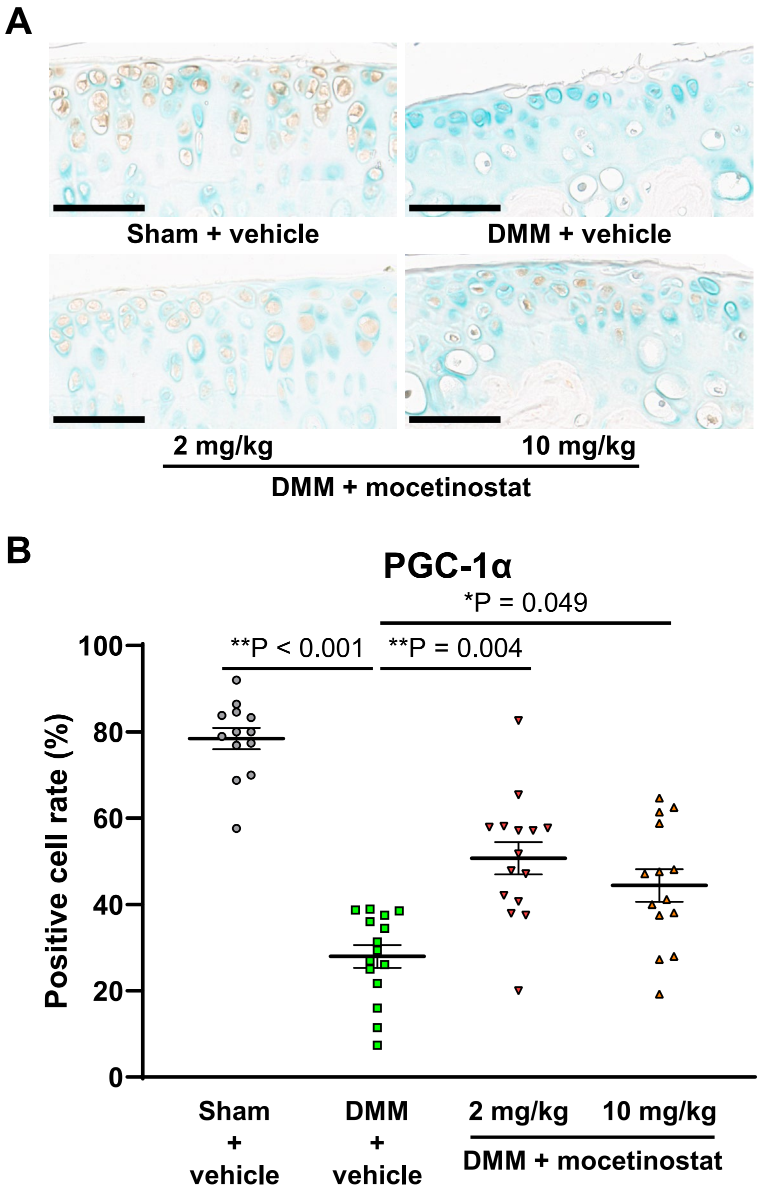

Supplemental Figure 16. PGC-1α expression in mocetinostat-treated mice after DMM surgery. This figure shows data related to the experiments in Figure 5. (**A**, **B**) Immunohistochemistry for PGC-1α in knee cartilage was performed. n=13 for Sham + vehicle, n=15 for DMM + vehicle and DMM + 2 mg/kg of mocetinostat, and n=14 for DMM + 10 mg/kg of mocetinostat. Scale bars, 60 μm. *P<0.05, **P<0.01, Dunn's test versus DMM + vehicle. All quantitative data are expressed as means±SE, and results of Kruskal-Wallis test are shown in Supplemental Table 17.

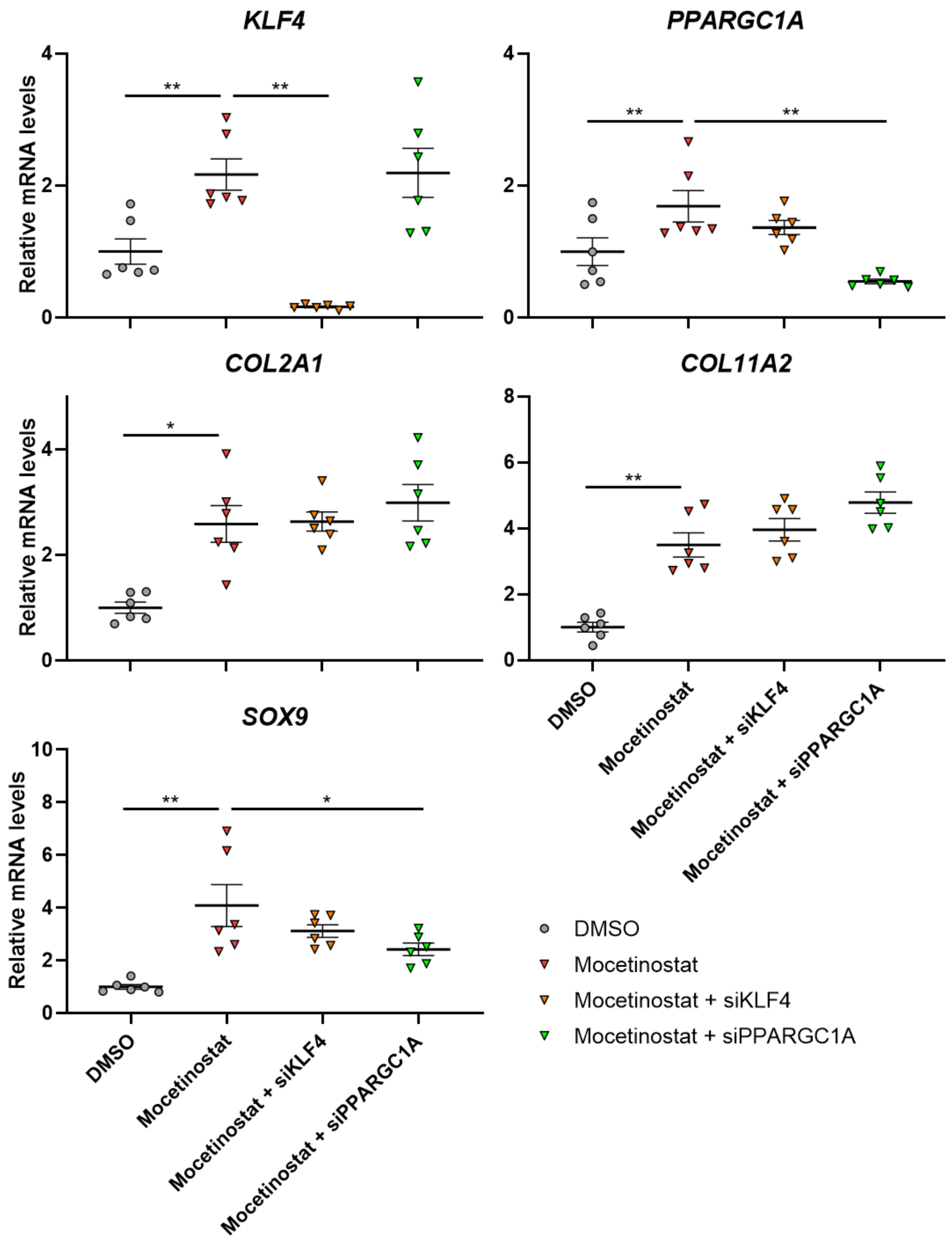

Supplemental Figure 17. siRNA knockdown of *KLF4* and *PPARGC1A* in mocetinostat-treated OA chondrocytes. Cells were transfected with siRNAs, and were treated with 30 μM mocetinostat or DMSO (n=6 donors). RNA was collected 24 hours after initiation of mocetinostat treatment. mRNA levels are expressed as means±SE, relative to DMSO. *P<0.05, **P<0.01, Dunnett's test versus mocetinostat. Results of one-way mixed-effects ANOVA test are shown in Supplemental Table 17.

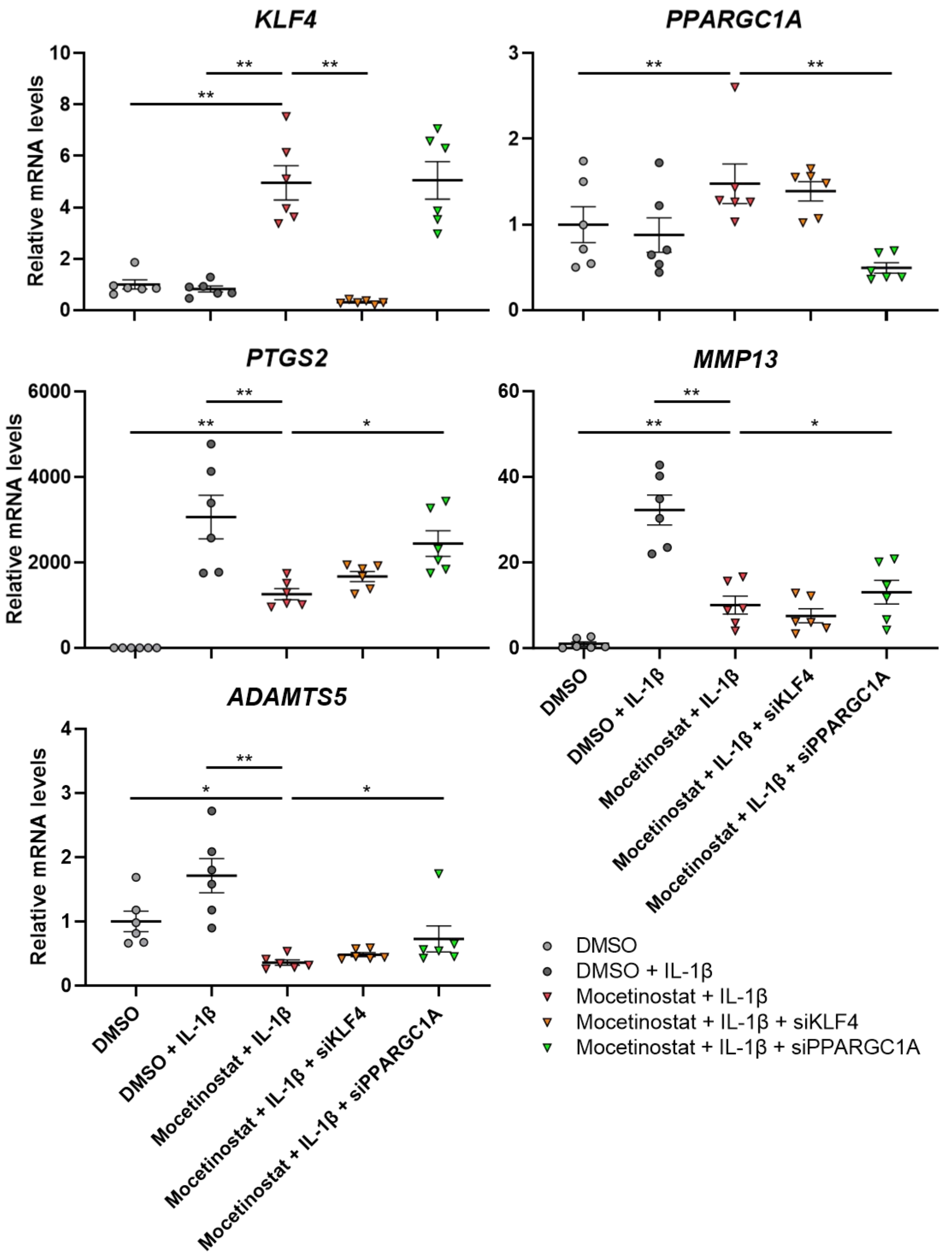

Supplemental Figure 18. siRNA knockdown of *KLF4* and *PPARGC1A* in mocetinostat-treated OA chondrocytes on IL-1β stimulation. Cells were transfected with small interfering RNAs (siRNAs), and were treated with 30 μM mocetinostat or DMSO (n=6 donors). RNA was collected 24 hours after initiation of mocetinostat treatment and 6 hours after stimulation with 10 ng/ml of IL-1β. mRNA levels are expressed as means±SE, relative to DMSO. *P<0.05, **P<0.01, Dunnett's test versus mocetinostat + IL-1β. Results of one-way mixed-effects ANOVA test are shown in Supplemental Table 17.

Supplemental Table 1. List of the hit compounds in the HTS.

Compound	EC_{50} (M)
Birinapant	1.29E-08
Bardoxolone methyl	1.95E-08
Dabrafenib mesylate	2.04E-08
Chidamide	2.50E-08
Cositecan	3.38E-08
AR-42	3.80E-08
Bortezomib	3.94E-08
Anisomycin	5.66E-08
Mocetinostat	5.76E-08
Pharmaprojects No. 6518	6.70E-08
PCA-4248	7.15E-08
Centbucridine	1.31E-07
CC-115	1.61E-07
PCI-24781	1.62E-07
Oprozomib	1.82E-07
XEN-723	1.86E-07
TVB-2640	2.19E-07
Antimycin A3	2.47E-07
9-methoxycamptothecin	3.59E-07
Actinomycin D	3.69E-07
Diclazuril	3.81E-07
FR-194738	4.40E-07
Pelitinib	4.46E-07
Belotecan hydrochloride	4.47E-07
Octenidine dihydrochloride	4.50E-07
Stilbazium iodide	4.85E-07
Pyrvinium pamoate	4.99E-07
Entinostat	5.49E-07
Carfilzomib	5.61E-07
Tosedostat	6.04E-07
SN-22	6.23E-07
Emetine	6.35E-07
Edotecarin	6.36E-07
Ixazomib citrate	6.52E-07
PRLX-93936	6.53E-07
IMD-0354	7.07E-07
Exatecan mesylate	7.23E-07
SN-38	7.33E-07
Adefovir dipivoxil	7.37E-07
APPCL	7.40E-07
56-472	7.55E-07
PF-05175157	7.99E-07
MK-8245	8.00E-07
Peruvoside	8.05E-07
ICI-73602	8.12E-07
PNU-166148	8.31E-07
RG-2833	8.43E-07
Delimotecan	8.61E-07
Narasin	8.70E-07
BW-114	9.04E-07
MKT-077	9.63E-07

Supplemental Table 2. List of the compounds confirmed in the secondary screen.

Compound	Classification	EC_{50} (M)
Tosedostat	Aminopeptidase inhibitor	1.66E-08
Entinostat	Class I Histone deacetylase inhibitor	1.78E-08
Peruvoside	Cardenolide glycoside	2.39E-08
9-methoxycamptothecin	Topoisomerase inhibitor	8.87E-07
TVB-2640	Fatty acid synthase inhibitor	3.83E-08
Dabrafenib mesylate	Raf kinase inhibitor	2.51E-07
Belotecan hydrochloride	Topoisomerase inhibitor	6.09E-07
Chidamide	Class I Histone deacetylase inhibitor	3.35E-07
PF-05175157	Acetyl-CoA carboxylase inhibitor	3.48E-07
Mocetinostat	Class I Histone deacetylase inhibitor	4.42E-07
ICI-73602	Antirhinovirus compound	4.68E-07
SN-38	Topoisomerase inhibitor	5.76E-07
Stilbazium iodide	Anthelmintic	5.16E-07
Oprozomib	Proteasome inhibitor	6.26E-07
XEN-723	Stearoyl-CoA desaturase inhibitor	6.36E-07
Octenidine dihydrochloride	Cationic surfactant with antimicrobial activity	8.38E-07
Exatecan mesylate	Topoisomerase inhibitor	8.50E-07
Emetine	Antiprotozoal	9.11E-07

Supplemental Table 3. Cell survival rates of SW1353 cells treated with the 18 identified compounds. Cells were treated with either of the identified compounds or DMSO for 24 hours. Each compound was used with different doses (20 nM, 50 nM, 100 nM, 200 nM, 500 nM, 1 μM, 2 μM, 5 μM, 10 μM, 20 μM and 30 μM), and cell survival rates of each compound were measured at the highest doses with normal cell viability (microscopically observed). Cell survival rates are expressed as means±SE (n=4 from four independent experiments). Dunn's test, versus DMSO. Results of Kruskal-Wallis test are shown in Supplemental Table 17.

Treatment	Concentration	Cell survival rates (%)	P values
DMSO	-	94.0±0.5	-
Tosedostat	30 μM	90.9±0.4	0.341
Entinostat	2 μM	91.1±0.4	0.561
Peruvoside	50 nM	90.5±0.8	0.209
9-methoxycamptothecin	10 μM	92.3±0.7	> 0.999
TVB-2640	30 μM	90.7±0.5	0.170
Dabrafenib mesylate	30 μM	92.1±0.6	> 0.999
Belotecan hydrochloride	2 μM	93.3±1.4	> 0.999
Chidamide	5 μM	94.2±0.9	> 0.999
PF-05175157	30 μM	93.2±0.9	> 0.999
Mocetinostat	2 μM	93.3±1.2	> 0.999
ICI-73602	500 nM	92.5±0.9	> 0.999
SN-38	10 μM	90.9±1.1	0.477
Stilbazium iodide	2 μM	90.4±0.7	0.119
Oprozomib	50 nM	90.9±1.1	0.497
XEN-723	30 μM	92.0±0.6	> 0.999
Octenidine dihydrochloride	2 μM	92.2±1.1	> 0.999
Exatecan mesylate	2 μM	93.0±0.6	> 0.999
Emetine	50 nM	91.2±2.1	> 0.999

Supplemental Table 4. Summary of experiments for SW1353 cells treated with the compounds upregulating *KLF4*. The results of statistical analyses are summarized for the experiments shown in Figure 1B. * and **, significant upregulation with $P<0.05$ and $P<0.01$, respectively. DR, significant downregulation. NS, non-significant changes. Dunnett's test versus DMSO (n=4 from four independent experiments). Results of one-way mixed-effects ANOVA test are shown in Supplemental Table 17.

Compound	*COL2A1*	*COL11A2*	*SOX9*	*ACAN*
Tosedostat	NS	NS	NS	NS
Entinostat	*	**	**	*
Peruvoside	NS	NS	NS	NS
9-methoxycamptothecin	NS	*	**	DR
Belotecan hydrochloride	NS	**	**	NS
Chidamide	**	**	**	*
PF-05175157	NS	NS	*	NS
Mocetinostat	**	**	**	*
SN-38	NS	**	**	NS
Stilbazium iodide	NS	**	NS	DR
Octenidine dihydrochloride	NS	NS	NS	DR
Exatecan mesylate	*	**	**	NS

Supplemental Table 13. Summary of enriched pathways common between URGs of RNA-seq and URPs of TMT-MS.

Enriched pathways common between URGs of RNA-seq and URPs of TMT-MS	URGs of RNA-seq for KLF4-overexpressed samples
Arrhythmogenic right ventricular cardiomyopathy	Enriched
PPAR signaling pathway	Not enriched
Dilated cardiomyopathy	Enriched
Hypertrophic cardiomyopathy	Enriched
Leukocyte transendothelial migration	Enriched
Arginine and proline metabolism	Not enriched
ECM-receptor interaction	Enriched
Proteoglycans in cancer	Enriched
Toxoplasmosis	Not enriched

Supplemental Table 14. Extracted data for PPARs and PPARGCs in RNA-seq of mocetinostat- versus DMSO-treated samples.

Gene ID	Gene symbol	Log2(FC)	*P* value	FDR
ENSG00000186951	PPARA	5.77E-01	2.75E-02	6.86E-02
ENSG00000112033	PPARD	2.29E-01	1.24E-01	2.32E-01
ENSG00000132170	PPARG	-4.70E-01	1.41E-02	3.92E-02
ENSG00000109819	PPARGC1A	5.08E+00	4.53E-24	1.38E-22
ENSG00000155846	PPARGC1B	3.57E-01	6.42E-02	1.38E-01

Supplemental Table 15. List of TaqMan probes.

Species	Gene	Probe
Human	*ACAN*	Hs00153936_m1
Human	*ADAMTS5*	Hs01095518_m1
Human	*COL1A1*	Hs00164004_m1
Human	*COL2A1*	Hs00264051_m1
Human	*COL10A1*	Hs00166657_m1
Human	*COL11A2*	Hs00365416_m1
Human	*FOXO1*	Hs00231106_m1
Human	*GAPDH*	Hs02786624_g1
Human	*KLF2*	Hs00360439_g1
Human	*KLF4*	Hs00358836_m1
Human	*IL6*	Hs00174131_m1
Human	*MMP3*	Hs00968305_m1
Human	*MMP13*	Hs00233992_m1
Human	*PPARGC1A*	Hs00173304_m1
Human	*PRG4*	Hs00981633_m1
Human	*PTGS2*	Hs00153133_m1
Human	*RUNX2*	Hs00231692_m1
Human	*SCX*	Hs03054634_g1
Human	*SOX9*	Hs00165814_m1
Human	*TNXB*	Hs00372889_g1

Supplemental Table 16. Antibodies used for IHC.

Antibody	Clone	Manufacturer (Cat. No.)	Dilution
Goat anti-KLF4	Polyclonal	R&D Systems (#AF3158)	1:300
Rabbit anti-PGC-1α	Polyclonal	Novus Biologicals (NBP1-04676)	1:200
Rabbit anti-FOXO1	C29H4	Cell Signaling Technology (#2880)	1:50
Rabbit anti-ADAMTS5	Polyclonal	Abcam (#ab41037)	1:100
Rabbit anti-IL6	Polyclonal	Abcam (#ab6672)	1:600
Rabbit anti-MMP13	Polyclonal	Abcam (#ab39012)	1:300
